# Subtelomeric plasticity contributes to gene family expansion in the human parasitic flatworm *Schistosoma mansoni*

**DOI:** 10.1186/s12864-024-10032-8

**Published:** 2024-02-27

**Authors:** T Brann, A Beltramini, C Chaparro, M Berriman, SR Doyle, AV Protasio

**Affiliations:** 1 Department of Pathology, University of Cambridge, Cambridge, CB1 2PQ UK; 2 IHPE, CNRS, IFREMER, UPVD, University Montpellier, Perpignan, F-66860 France; 3 School of Infection and Immunity, University of Glasgow, Glasgow, G12 8TA UK; 4 Wellcome Sanger Institute, Cambridge, CB10 1SA UK; 5 Christ’s College, Cambridge, CB2 3BU UK

**Keywords:** *Schistosoma mansoni*, Transposable element, Genomic repeats, Subtelomere, Non-allelic homologous recombination, Segmental duplication

## Abstract

**Background:**

The genomic region that lies between the telomere and chromosome body, termed the subtelomere, is heterochromatic, repeat-rich, and frequently undergoes rearrangement. Within this region, large-scale structural changes enable gene diversification, and, as such, large multicopy gene families are often found at the subtelomere. In some parasites, genes associated with proliferation, invasion, and survival are often found in these regions, where they benefit from the subtelomere's highly plastic, rapidly changing nature. The increasing availability of complete (or near complete) parasite genomes provides an opportunity to investigate these typically poorly defined and overlooked genomic regions and potentially reveal relevant gene families necessary for the parasite’s lifestyle.

**Results:**

Using the latest chromosome-scale genome assembly and hallmark repeat richness observed at chromosome termini, we have identified and characterised the subtelomeres of *Schistosoma mansoni*, a metazoan parasitic flatworm that infects over 250 million people worldwide. Approximately 12% of the *S. mansoni* genome is classified as subtelomeric, and, in line with other organisms, we find these regions to be gene-poor but rich in transposable elements. We find that *S. mansoni* subtelomeres have undergone extensive interchromosomal recombination and that these sites disproportionately contribute to the 2.3% of the genome derived from segmental duplications. This recombination has led to the expansion of subtelomeric gene clusters containing 103 genes, including the immunomodulatory annexins and other gene families with unknown roles. The largest of these is a 49-copy plexin domain-containing protein cluster, exclusively expressed in the tegument—the tissue located at the host-parasite physical interface—of intramolluscan life stages.

**Conclusions:**

We propose that subtelomeric regions act as a genomic playground for trial-and-error of gene duplication and subsequent divergence. Owing to the importance of subtelomeric genes in other parasites, gene families implicated in this subtelomeric expansion within *S. mansoni* warrant further characterisation for a potential role in parasitism.

**Supplementary Information:**

The online version contains supplementary material available at 10.1186/s12864-024-10032-8.

## Introduction

The subtelomere is the region immediately adjacent to the telomere within a chromosome. Subtelomeres have a high propensity for non-allelic homologous recombination, resulting in segmental duplications (SDs), a phenomenon stimulated by repetitive elements [1]. This results in the expansion of taxa-specific gene families often associated with phenotypic variability and makes the subtelomere a rapidly changing interface and potent contributor to genome evolution [2–4].

Despite the subtelomeres’ evolutionary importance, these are poorly described to date, particularly in non-model organisms. Technical limitations in sequencing approaches and the intrinsic repetitiveness of these regions have left subtelomeres mostly unassembled and are, therefore, poorly represented and subsequently neglected in many genomes [5, 6]. Fortunately, recent advances in long-read sequencing and HiC-assisted scaffolding have afforded telomere-to-telomere and chromosomal level assemblies in many organisms, which provides new insights into repetitive regions, such as subtelomeres [7, 8]. For example, in the new telomere-to-telomere human genome, these technologies revealed ten times more SDs than previously identified, predominantly across newly assembled chromosome ends [9]. Until recently, genome sequencing has been prohibitively expensive and resource intensive [10] and parasite genomes, including subtelomeres, assembled during this time are highly fragmented [11].

Subtelomeres are particularly pertinent in parasitology research, as multi-copy genes found in these regions are often associated with parasite invasion, proliferation and fitness [12]. For example, in two important human parasites *Trypanosoma brucei* and *Plasmodium falciparum*, virulence factors (variant surface glycoprotein & *var* genes*,* respectively) that help evade the host immune system are encoded by subtelomeric multi-copy gene families [12, 13]. The presence of many copies of these genes, generated by subtelomeric recombination facilitates the switching of immunogenic epitopes, prolonging immune evasion and, in turn increasing parasite fitness and transmission [14, 15].

*Schistosoma mansoni* is the best-characterised species of the Schistosomatidae family of parasitic flatworms. Schistosomes are the causative agents of schistosomiasis, or bilharzia, a parasitic disease affecting approximately 250 million people, mainly in lower and middle-income countries [16, 17]. The life cycle of schistosomes alternates between freshwater snails, as intermediate hosts, and a definitive vertebrate host, in which sexual reproduction occurs. Within humans, untreated infections become chronic persisting for decades within the host’s vasculature [18]. Long-term survival in this hostile environment is associated with complex, but not fully characterised, immune evasion strategies in an ongoing evolutionary arms race between host and worm [19, 20]. Given that schistosomes have a comprehensive ability to modulate the host immune system and that immunomodulatory genes in other parasites (*e.g.* trypanosomes and *P. falciparum*) are found at the subtelomere, these genomic regions may be similarly important in schistosomes.

The genome of *S. mansoni* has a high repeat content, with approximately 35% attributed to transposable elements (TE) and about 10% occupied by tandem repeats and *Schistosoma*-specific repeats, known as “W Elements” (WE) [21]. Repeats such as these are thought to stimulate SD formation and are a common feature of subtelomeres [1]. SDs have previously been explored in *S. mansoni*, where approximately 0.22% of the genome was identified as SD-derived with no specific genomic distribution or association with repetitive elements [22]. However, this analysis used a highly fragmented and partially assembled (non-telomere-to-telomere) genome. With the recent availability of an improved, near-complete telomere-to-telomere genome for *S. mansoni* [23], we aimed to identify the subtelomeres and characterise their content. Understanding these genomic regions may provide insight into genes and gene families that exploit the highly plastic nature of the region and the evolutionary mechanisms that allow *S. mansoni* to parasitise its host.

## Methods

### Genome annotation and genome coverage calculations

Repeats were annotated in version nine of the *S. mansoni* genome (PRJEA36577, GCA_000237925.5, WormBase ParaSite release WBPS17) [23] using manually curated libraries of *S. mansoni* TE and WE [21] sequences as separate inputs into RepeatMasker v4.1.236 [24] with parameters: "-no_is -gff -s -a". The genomic locations of mapped TEs can be found in Supplementary Table 1. RepeatMasker uses tandem repeat finder (TRF) [25] to identify tandem/simple repeats, which were also collected. Repeat coverage of TEs and other repeats (a combination of WEs and simple repeats/tandem repeats) was quantified using bedtools coverage [26] in 1 Mb windows of the *S. mansoni* genome with 100 kb steps. GC content was determined in 500 kb non-overlapping windows across the genome using bedtools nuc. Repeat- and GC-content was visualised using the circlize [27] and MetBrewer [28] packages implemented in R (v2022.07.01) [29]. Gene content analyses were done taking the longest transcript isoform per gene (Supplementary File 1).

### Definition of subtelomere

Subtelomeric regions were defined by identifying the first region of repetitive element enrichment at both ends of each chromosome, using the combined repeat density (*i.e.* TEs, WEs, tandem repeats, but excluding telomeric repeats) in 1 Mb windows with 100 kb step. *S. mansoni* autosomes are resolved into single scaffolds; however, the Z and W sex-chromosomes are divided into separate regions in the assembly file used, which includes two pseudoautosomal regions (PAR1 and PAR2) common to Z and W, and Z-specific and W-specific regions unique to Z and W, respectively. Because we aimed to define subtelomeres for all chromosome ends, we artificially joined PAR1 and PAR2 into a single scaffold termed "Merged PARs".

Repeat element density was non-normally distributed (*p* < 0.001; Shapiro–Wilk test), and, therefore, a one-tailed Wilcoxon test was used to calculate a *p*-value per window across chromosomes. A Savitsky-Golay filter [30], implemented in the Signals package for R [31] was used to denoise the *p*-values. The filter requires two parameters, “p” (set to 1) and “n” (set to 1/35th of the length of the dataset), in addition to the *p*-values of a given chromosome (for further details, see bioinformatics workbook, Supplementary File 2). Subtelomeric bounds were automatically identified from the denoised *p*-values using auto_sub.py (https://github.com/tbrann99/Subtelomere). Subtelomere coordinates were then adjusted to remove regions of telomeric repeats present on the chromosome, as identified by tidk search, using 100 bp windows [32]. Subtelomere coordinates with the most recent genome assembly (assembly SM_V10 in WormBase ParaSite release WBPS18) are provided for forwards compatibility **(**Supplementary File 3 & Supplementary Table 2).

Gene, TE and other repeat densities in subtelomeres and chromosome bodies were calculated using bedtools coverage [26]. A single density value was reported for each given chromosomal region, and sex-specific regions were excluded from the ‘chromosome body’ quantification. After testing for normality with a Shapiro–Wilk test, a two-sample t-test was used to compare differences between “chromosome body” and “subtelomere” region content.

### Identification of segmental duplications

Whole genome sequence homology blocks were identified using BISER (v1.3) [33], a specialised tool for SD identification, on an unmasked *S. mansoni* genome [23] with default parameters except for "--temp --keep-contigs --keep-temp". Hits were filtered to remove intrachromosomal SDs (treating PAR1/PAR2 as separate chromosomes), SDs derived from sex-specific regions, hits under 20 kb in length, and hits that were more than 80% combined repeat content. Repeat content was calculated across all repeat types using bedtools coverage [26], combining TE, WE (a *Schistosoma*-specific tandem repeat [21], tandem repeats, manually annotated repeats, telomeres, and centromere repeats. Manually annotated repeats include 36 loci where large tandem repeats had been incompletely annotated by TRF and were verified with dot plots [34] and manually annotated to identify new repeat bounds (Supplementary File 4 & Supplementary Table 3). We then filtered the remaining hits to remove low-quality alignments using BISER’s total alignment error metric, requiring a value lower than 70% total alignment error. SDs were then categorised as subtelomeric—where at least one locus within the pair was subtelomeric; or chromosome body, where both loci fell within the chromosome body (classified by subtel.py, https://github.com/tbrann99/Subtelomere). Hits were visualised in a circular plot, in which the ideogram was annotated with subtelomere bounds. Segmental duplication coverage for respective regions was calculated using bedtools coverage and compared using a Chi-squared test, in which genomic regions were stratified in regard to SD coverage; higher or lower than the median. Expected values were generated assuming a random distribution of SD-rich regions across regions sampled (Supplementary Table 4). A Chi-squared test was also used to assess number of SDs (as opposed to coverage). Expected values for the number of SDs occurring in different chromosomal regions was once again derived from a random distribution of SDs around the genome, excluding the Z and W specific regions (Supplementary Table 4). Pairwise nucleotide distance for segmental duplications was calculated with the EMBOSS distmat function and the Jukes Cantor substitution model [35] and represented in a swarm plot [36]. 

### Segmental duplication content

Overlaps between mRNA annotations and SDs were identified using bedtools intersect [26] with default parameters and a modified annotation file (only the longest splicing isoform, where relevant, was considered—Supplementary File 1) and the SD annotation file (Supplementary Table 5). A sequence similarity network (SSN) [37] was generated using similarity scores derived from pairwise comparisons of protein sequences (predicted peptides can be found in Supplementary File 5). A 30% amino acid identity threshold yielded 11 clusters (four having two genes each). SSN was visualised in Cytoscape v3.9.1 [38] and coloured by region (subtelomere or chromosome body). Product tags were added to clusters based on pre-existing functional annotations in WormBase ParaSite release WBPS17 (Supplementary Table 6) except for the ‘hypothetical protein’ cluster, for which most genes lack a product description. Based on our results (see next section) we later renamed them plexin domain-containing protein. Chromomap [39] was used to display distribution of segmentally duplicated gene clusters in subtelomere. Density of simple repeats, TEs and WEs was assessed in each of the 1,936 SDs and the adjacent 2 kb in 100 base windows across the genome using Deeptools [40]. Repetitive elements were plotted separately to test for enrichment of individual repeats surrounding SDs. 

### Gene expression

Bulk RNA-Seq data covering various stages of the *S. mansoni* life cycle [41] was used to investigate expression of selected subtelomeric gene clusters identified from the previous section. Of the time points available from Buddenborg et al*.* (2023), five replicates of mixed sex stages of eggs, miracidia, 1-day sporocyst, 5-day sporocyst, 32-day sporocyst, cercariae, 2-day schistosomula were used. In addition, five replicates of male and female 26-day post-infection juvenile worms were used. Sequencing reads were aligned to *S. mansoni* version 9 assembly GCA_000237925.5 [23] using STAR (v2.7.8) [42] with outFilterMultimapNmax flag set to 100. This ensures that multi-mapping reads, which may arise from multicopy gene families, are not discarded under the default parameters. Reads per transcript were counted using the featureCounts function from subread (v2.0.3) [43] with default parameters except for "--primary -t exon -g transcript_id" and the pre-release gene annotation (Supplementary File 1). The primary flag ensures that each read is counted only once from potentially multi-mapped reads. We processed counts by calculating Transcripts per Million (TPM, Supplementary Table 7) [44] and then normalising to a Z-score per gene across life stages using the pheatmap package [45]. Differential expression analyses between relevant life stages were performed using DESeq2 (v1.36.0) [46]. Transcript with a read count < 10 reads were removed, and log-fold change shrinkage was conducted with apeglm estimation [47]; these data were represented in MA plots. For this comparison, 1-, 5-, and 32-day sporocyst samples were aggregated into a single “Sporocyst” sample and the schistosomula and juvenile samples into an “Intra-mammalian” sample. 

### sc-RNASeq analysis

Publicly available single-cell RNA-seq (scRNA-seq) data [48–50] was reanalysed in the context of selected subtelomeric gene clusters. Samples used include two mixed-sex miracidia collected within four hours of hatching [48], four replicates of five 5-day-old sporocysts [49] and four replicates of three male and three female worms [50]. Analysis was completed using the equivalent methodology and parameters used in the respective associated publications with minor changes described below. Sequencing reads were mapped to the *S. mansoni* genome (assembly version 10) and gene annotation available in WormBase ParaSite release WBPS18 [51] using cellranger 7.0.1 [52]. Although a newer genome version was used, gene annotations remain largely unchanged (Supplementary File 3 and Supplementary Table 2). Analysis on mapped reads was performed using Seurat [53]. Quality control, including filtering based on percentage of mitochondrial genes, number of transcripts, and number of genes identified per cell (Supplementary Table 8), was done separately for each dataset and biological sample and subsequently integrated. Sporocyst samples had fewer cells, and therefore, less stringent filtering was applied. In these cases, scDblFinder (v1.14.0) [54] (https://github.com/plger/scDblFinder) with default parameters was used to remove multiplets (multiple cells in a single droplet). Elbow plots and clustree [55] were used to identify optimal number of principal component elements and resolution of the Louvain algorithm, respectively. 

Marker genes were identified by ROC (Receiver Operating Characteristic) analysis and were used, alongside manual filtering of undistinguished clusters and data from the original publications [48–50], for cluster identification (Supplementary Table 8). To ease visualisation in pheatmap [45], cell type clusters were simplified by aggregating them by classification (*e.g.* in adult life stages, neuron clusters 1–35 were reclassified into a single “neuron” cluster, detailed list, see Supplementary Table 8) and a mean read count across merged cell types was used. scRNA counts per cell were scaled per row per dataset using the Seurat function ScaleData [53]. 

### Characterising the ‘hypothetical protein’ cluster

Hypothetical protein gene models were visualised with gggenes (v0.5.0) [56] and ggplot2 [57] packages implemented in R [29]. Gene_viz.py (https://github.com/tbrann99/Subtelomere) was used to reformat the annotation (Supplementary File 1) for the gggenes package. Exons were coloured by their order of appearance in the transcript and aligned by their start codon for all sequences except seven models lacking the canonical first two exons. These additional seven genes were aligned at the third exon position and coloured to reflect exon number plus two, demonstrating their similarity to the ‘full length’ gene models. All gene models were represented in the direction of transcription.

Hypothetical protein sequences were aligned using muscle [58] (v5.1) (Supplementary File 6) with default parameters and a dendrogram was constructed from this alignment using IQ-Tree [59] with 1000 iterations of ultrafast bootstrapping [60]. The resulting tree file was visualised on the Interactive Tree of Life (ITOL) [61] web tool. Conserved protein domains were identified using InterProScan5 Representational State Transfer API [62], and the resulting tsv annotation file was processed with anno_domain_viz.py (https://github.com/tbrann99/Subtelomere) to generate ITOL-compatible protein domain annotations. Phobius [63] annotations were redundant with those from TmHelix (TMHMM2.0) [64] and were removed. 

The previously generated multiple sequence alignments were used to search a protein database of reference proteomes using hmmsearch (HmmerWeb version 2.41.2 [65], with default parameters. Briefly, hmmsearch uses a protein alignment or profile Hidden Markov model (HMM) as a query and performs a homology search against a protein database. Using this approach versus a single sequence homology search improves sensitivity and improves identification of conserved residues across more distantly related sequences. Alignments to a Plexin-Semaphorin-Integrin (PSI) domain (PF01437) were visualised using the previously generated amino acid alignment in Jalview [66], with residues coloured by “clustal” if the percentage identity was greater than 80%. Sequences with large gaps or that were fragmented were removed from the visualisation. Five prime signal peptide and 3’ transmembrane domains were partially trimmed. A residue error plot provided by AlphaFold (DB version 2022-11-01) [67, 68] for Smp_173350.1 (one of the few full-length *S. mansoni* hypothetical proteins with a putative PSI-domain included in Alphafold, due to the very recent annotation of a majority of subtelomeric genes) was visualised alongside putative domains to identify resolved structures. The protein’s 3D structure was visualised in Pymol (2.4.2) [69]. 

Additional members of this gene family were identified using the previously described clustering methodology (30% amino acid identity) and all *S. mansoni* amino acid sequences, as opposed to just those segmentally duplicated (Supplementary Table 9).

## Results

### Defining *Schistosoma mansoni*'s subtelomeres

**Fig. 1:**
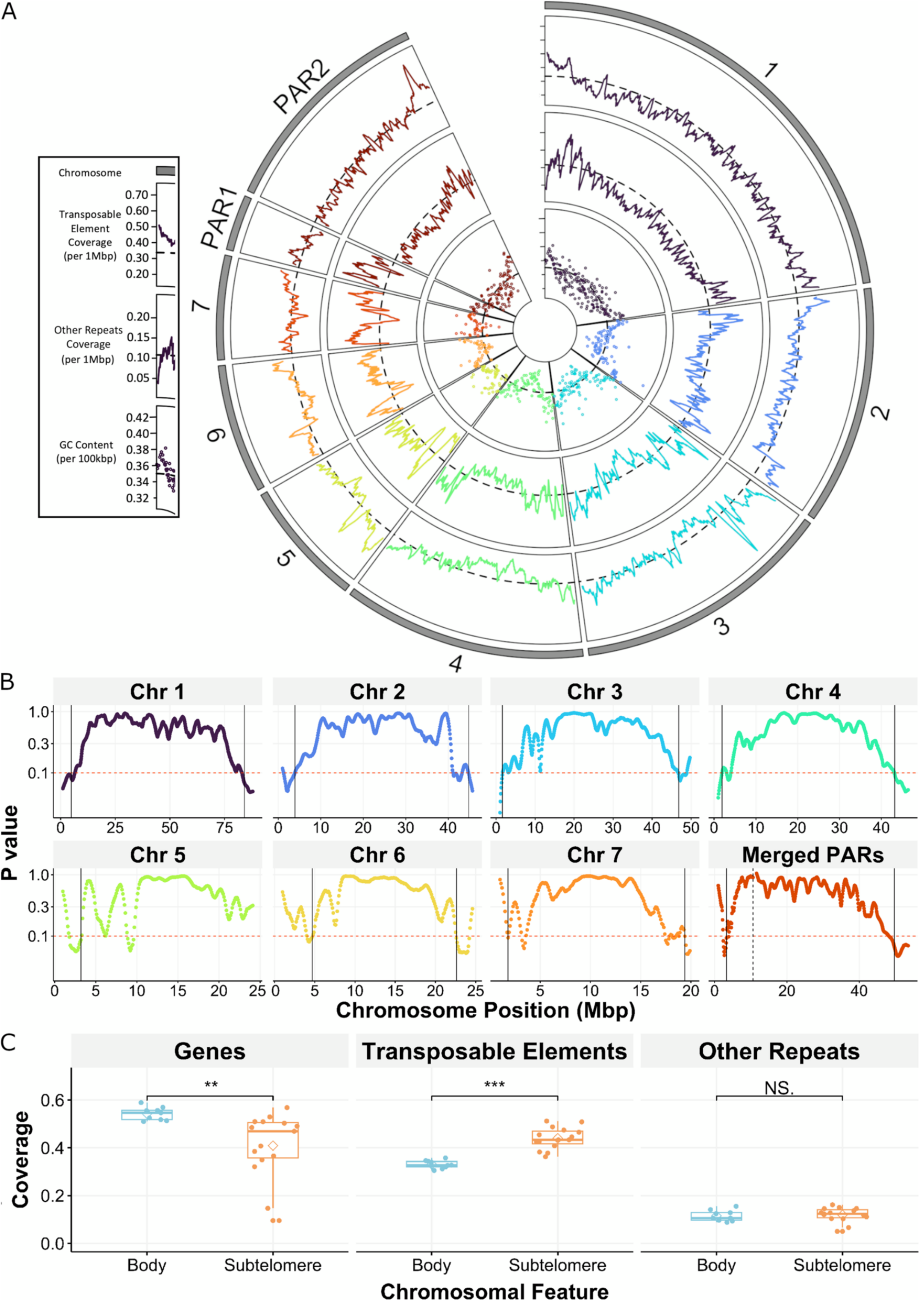
Defining subtelomeres at repeat-rich *Schistosoma mansoni* chromosome termini. **A**) Circular plot representing features coverage across seven autosomes (1–7), and two pseudoautosomal regions (PAR1 and PAR2) shared by Z and W sex chromosomes. Outer and middle rings: Transposable Elements and other repetitive elements (excluding telomeric repeats) respectively, plotted across 1 Mb windows, with 100 kb non-overlapping steps. Inner ring: GC content plotted as single data points in non-overlapping 500 kb windows.. Dashed black lines indicate the genomic means. **B**) Patterns of combined repeat density observed at chromosome termini were used to statistically define subtelomeres using the same repetitive element windows described in A. Boundaries between subtelomere and chromosome body are indicated with black vertical lines. All but the right-hand end of Chromosome 5 reached the threshold (red dashed line, *p* = 0.1; one-tailed Wilcoxon test) for subtelomere definition. Two pseudoautosomal regions (PAR1 and PAR2) that are shared by the sex chromosomes have been artificially joined, with a black dashed line indicating the breakpoint between PAR1 (left) and PAR2 (right). Data processed with a Savitsky-Golay filter. **C**) Gene, transposable elements and other repeats coverage (as nucleotides) in newly defined subtelomeres and resultant chromosome body regions. Differences in coverage between subtelomere and chromosome body were assessed using a t-test (NS *p* > 0.05, ** *p* < 0.01, *** *p* < 0.001)

To identify regions of repeat element enrichment that may represent subtelomere-like regions in *S. mansoni*, we mapped a library of manually curated TEs, W elements and other repeats to version nine of the *S. mansoni* genome (GCA_000237925.5) [23]. The resulting comprehensive repeat map revealed an uneven distribution of repeats across all chromosomes. We observed that GC content and repeat density tend to be higher at chromosome termini (Fig. 1A, Supplementary Fig. 1). This pattern is also observed in pseudoautosomal regions, where the left-hand side of PAR1 and the right-hand side of PAR2 represent the telomeric ends of the sex chromosomes.

We hypothesised that we could use repeat element density to systematically define subtelomeric regions. To do so, we used a “one-tailed Wilcoxon test” of combined repeat density over 1 Mb windows (*p* = 0.1 threshold; Fig. 1B) and compared it to the chromosome’s mean repeat density. These signals were then denoised with a Savitsky-Golay filter. With this method, we classified *S. mansoni* subtelomeric regions based on enrichment of all repetitive elements (excluding telomeric repeats) observed at chromosome ends (Table 1). Statistical significance was not reached at the right end of Chromosome 5, and as such, no subtelomeric region was defined.

**Table 1:** Subtelomeric bounds and respective lengths, as defined by repeat richness. (*) Coordinates for the updated version 10 of the genome are provided in Supplementary Table 2

Chromosome (*) GCA_000237925.5	Start	End	Length	Start	End	Length
SM_V9_1	1	4,800,000	4,799,999	84,100,000	87,980,433	3,880,433
SM_V9_2	1	3,900,000	3,899,999	44,800,000	45,707,394	907,394
SM_V9_3	5,072	1,600,000	1,594,928	46,800,000	49,788,034	2,988,034
SM_V9_4	1	1,900,000	1,899,999	43,200,000	46,470,858	3,270,858
SM_V9_5	6,472	3,200,000	3,193,528	Not found	Not found	Not found
SM_V9_6	766	4,700,000	4,699,234	22,600,000	24,679,264	2,079,264
SM_V9_7	148	1,800,000	1,799,852	19,400,000	19,942,475	542,475
SM_V9_PAR1	1,356	3,300,000	3,298,644	n/a	n/a	n/a
SM_V9_PAR2	n/a	n/a	n/a	39,300,000	42,949,100	3,649,100

To determine which type of repeat element most prominently contributes to subtelomeres' repetitiveness, we performed an enrichment test of other repeats (including WEs and tandem repeats) and TEs (Fig. 1C) in subtelomeres and chromosome bodies. We found that TEs are enriched in these regions (*p* < 0.001; t-test) while other repeats are not, indicating that TEs are the main contributor to repetitiveness in these regions. As low gene density is another hallmark of subtelomeres [70], we also tested gene content of *S. mansoni*’s subtelomeres, and found that the mean fraction of nucleotides annotated as genes is statistically different between subtelomeres and chromosome bodies (t-test *p* < 0.01, Fig. 1C) suggesting less density of genes in the former.

### *S. mansoni*’s subtelomeres contribute to inter-chromosomal segmental duplications.

**Fig. 2:**
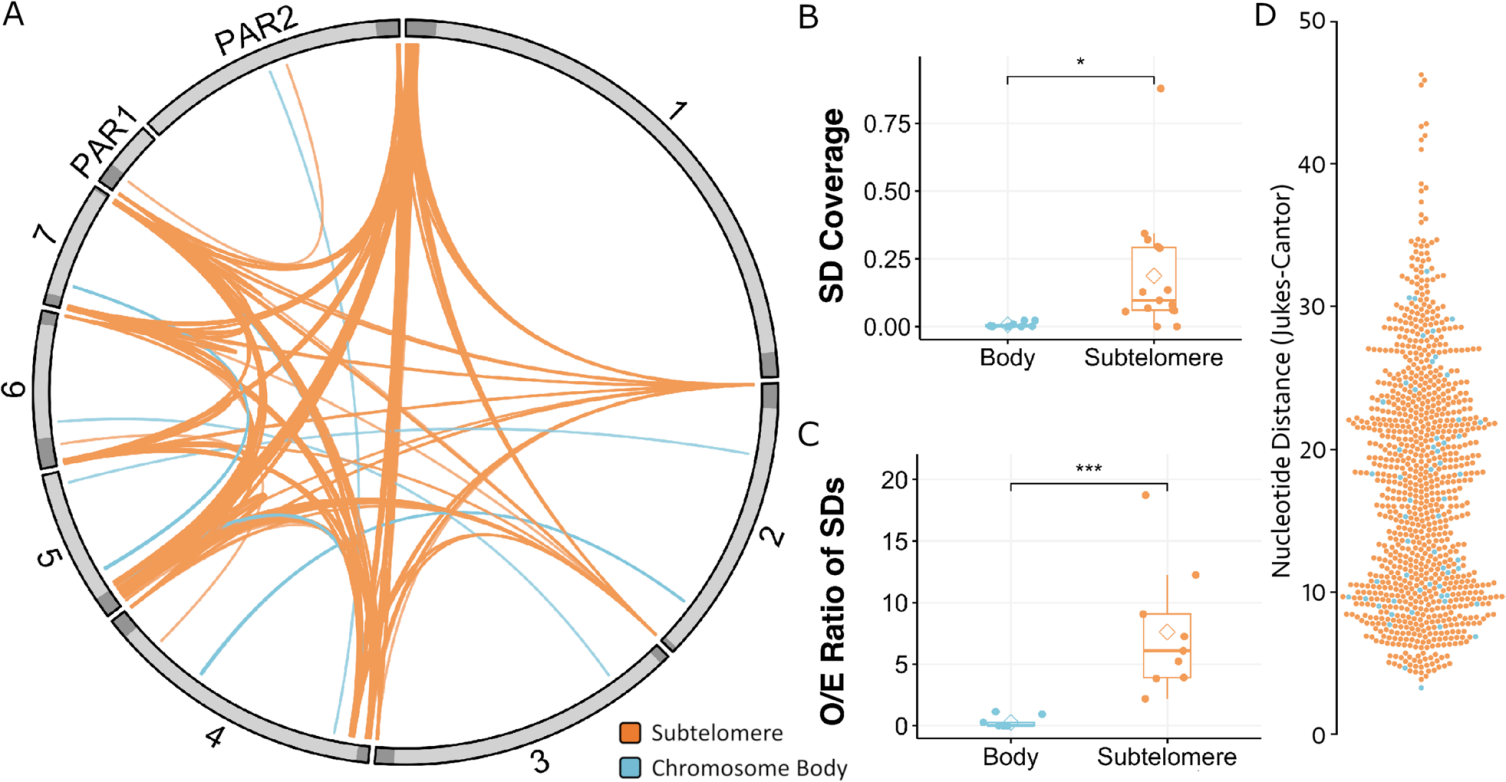
*Schistosoma mansoni*’s subtelomeres contribute to interchromosomal segmental duplications. **A**) Chromosomal map of segmental duplications (SD) highlighting regions of shared sequence similarity between *S. mansoni* chromosomes. SDs were classified as subtelomeric (if at least one of the two loci is subtelomeric, orange) or chromosome body (blue). The circle represents seven autosomal chromosomes (1–7) and two pseudoautosomal regions (PAR1 and PAR2). Chromosome bodies are shown in light grey, while subtelomeres are in dark grey. **B**) Differences in SD coverage for chromosome body and subtelomere of each chromosome were assessed (chi-squared test; * *p* < 0.05). **C**) The number of SDs in these regions was evaluated, generating an observed (O) vs. expected (E) ratio normalised by region size for each given chromosomal region, per chromosome (chi-squared test; *** *p* < 0.001). **D**) Nucleotide distance (Jukes-Cantor substitution model) of segmental duplications coloured by location

A hallmark of subtelomeres is their tendency to undergo segmental duplications (SDs) [71]. We used BISER [33], a specialised SD identification tool, to find instances of sequence similarity between chromosomes in the *S. mansoni* genome. We identify 968 non-repeat derived pairs (by excluding SDs with more than 80% combined repeat content) of homologous sequences > 20 kb, totalling approximately 2.3% of the *S. mansoni* genome. Of these pairs, 93.9% are associated with a subtelomere at one locus (*n* = 293) or both loci (*n* = 616), leaving 59 pairs falling in chromosome bodies (Fig. 2A). SDs are present at 13 of the 15 defined subtelomeres (not present at one end of chromosomes 1 and 3). We validated this observed enrichment of subtelomeric SDs in two ways. Firstly, we compared total SD coverage over newly defined subtelomeres and chromosome bodies (Fig. 2B, X^2^ = 7.2, *p* = 0.02), and, secondly, we analysed number of SDs observed in these regions (Fig. 2C, X^2^ = 10,931, *p* < 0.001). From these tests, we conclude that subtelomeres are statistically enriched in SDs when compared to chromosome bodies. Of all subtelomeres assessed, chromosome 7 has the largest SD coverage of 0.32 and 0.88 for left and right subtelomeres, respectively (*i.e.* 32% and 88% of subtelomeres are occupied by segmental duplications), compared to an all-subtelomere and chromosome body medians of 0.097 0.001 respectively. Chromosome 7’s subtelomeres had an SD O/E ratio of 18.7 compared to subtelomere and chromosome body medians of 6.08 and 0.01 respectively.

We were interested in finding out whether these SDs occurred relatively recent in time. To this end, we calculated the nucleotide distance (Jukes-Cantor) between SDs (Fig. 2D) and found a median of 17.33, ranging from 3.29 to 46.25 suggesting that these duplications have occurred over a long period of time and none of them have occurred recently enough as to not have accumulated at least one nucleotide difference (no SDs with nucleotide distance of 0).

### Subtelomeric segmental duplications encode large copy-number gene families and stage- and cell- specific expression.

**Fig. 3:**
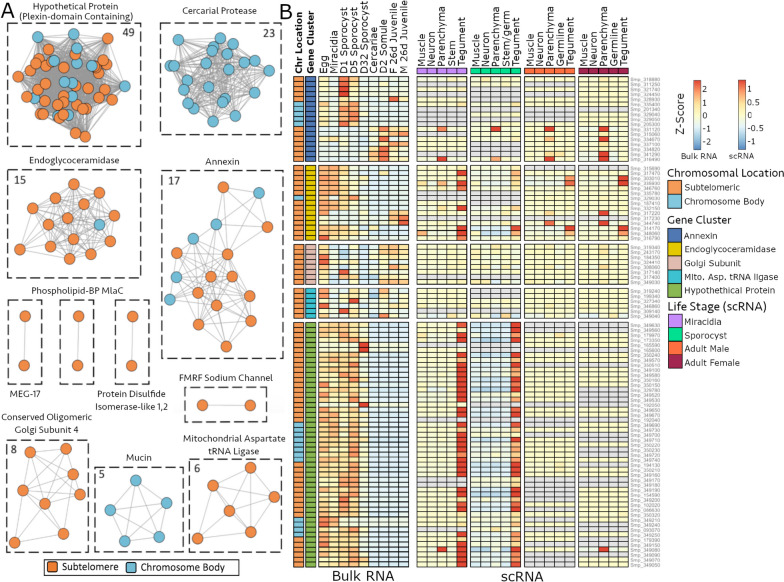
Segmental duplications have resulted in gene family expansion across *Schistosoma mansoni* subtelomeres. **A**) Sequence homology clustering of proteins encoded in SDs identifies discrete gene families amplified by SDs. Gene nodes are coloured by loci (orange for subtelomeres, blue for chromosome body), and number of genes per cluster and putative annotation are also shown. **B**) Heatmap representing relative transcript abundance (normalised to Z-score, by row and separately for each method) of expanded subtelomeric gene families analysed across multiple life cycle stages of *S. mansoni*. Bulk RNA-seq data [41] was sampled at the following time points and life stages: Eggs, Miracidia, Sporocyst D1 & D5 (mechanically transformed sporocysts recovered after 1 and 5 days of in vitro culturing), Sporocyst D32 (parasites recovered from in vivo snail infections 32 days post infection), cercariae, schistosomulae D2 (mechanically transformed schistosomula recovered after 2 days in in vitro culturing) and juvenile D26 male and female worms (recovered after 26 days post mouse infection). scRNA-seq analysis of Miracidia [48], Sporocyst (mechanically transformed and recovered after 5 days of in vitro culture) [49] and Male and Female adults [50]. Datasets were repurposed to analyse subtelomeric genes. Gene clusters shown are those with > two genes and found predominantly subtelomeric. Grey boxes indicate no detectable expression. Raw data used to generate heatmap can be found in Supplementary Table 7

When SDs are generated, any gene or genomic feature encoded in their sequence would also be duplicated in the genome. To investigate potential gene family expansions that resulted from SD, we overlapped gene annotation data and our SD coordinates and identified 154 genes falling in this category. Using an amino acid sequence similarity network approach, we defined discrete gene clusters that, given their high sequence similarity, could have a putative shared function (Fig. 3A, Supplementary Table 6). With this approach we describe clusters of “hypothetical proteins'' (*n* = 49; later renamed as plexin domain-containing proteins—see next section), annexins (*n* = 17) and endoglycoceramidases (*n* = 15), whose gene members are expanded across subtelomeres and to a lesser extent, into the chromosome body. Smaller clusters are entirely subtelomeric, including conserved oligomeric golgi subunit 4 (*n* = 8) and mitochondrial aspartate tRNA ligase (*n* = 6). We identified two clusters corresponding to cercarial proteases (*n* = 23) and mucins (*n* = 5), but these are not subtelomeric and were not analysed further (Supplementary Fig. 2). Finally, four sets of gene pairs were identified, and 23 singletons did not have sufficient amino acid similarity to any other genes in the dataset. SD genes; relative localisation with respect to chromosome termini and each other (Supplementary Fig. 3) revealed a slight tendency for clusters to be located closer to the chromosome termini. The distribution of repeats around segmental duplications was also visualised and no enrichment at or around breakpoints was observed (Supplementary Fig. 4).

To shed light on potential biological functions of the 103 genes that form subtelomerically expanded gene clusters, we analysed the expression profile of larger clusters (*n* > 2) using two different but complementary approaches (Fig. 3B). We investigated gene expression patterns over the parasites’ multi-stage life cycle by re-analysing previously published whole organism transcriptome (bulk RNAseq) data available for intra-molluscan, free-living and intra-mammalian stages. We complemented these data with single-cell RNA transcriptome data (scRNAseq, Supplementary Table 8) and gained cell-level resolution on the expression patterns of these subtelomeric clustered genes.

Whole organism transcriptome analysis suggests that no gene in any of these clusters seems constitutively expressed across the life cycle of these parasites. In fact, each gene shows higher expression at discrete stages. For example, the annexins show a group of nine genes with significantly higher expression in early sporocysts and another group with higher expression in intra-mammalian (schistosomula and juvenile) (Supplementary Fig. 5A). Similarly, 11 endoglycoceramidases are more highly expressed in egg stages, as opposed to miracidia stages (Supplementary Fig. 5B). On the other hand, all 49 genes in the hypothetical protein cluster are more expressed in eggs, miracidia and intra-molluscan stages, with log2-fold change values ranging from 4.4 to 16.1 when compared to intra-mammalian stages (Supplementary Fig. 5C, Supplementary Table 10). Temporal analysis of gene expression was complemented with cell specific gene expression by repurposing previously published scRNAseq datasets [48–50]. Many of the gene clusters with pronounced expression in specific life stages, did not show cell-type-specific expression; for instance, just four annexin genes were upregulated in parenchyma, with the remainder showing constitutive expression across many cell types. Between three and seven endoglycoceramidases were largely expressed in tegumental cells of miracidia, early sporocyst and adults but, more strikingly, the hypothetical protein cluster that is more highly expressed in expressed in egg, miracidia, and sporocyst stages (as evidenced in the bulk RNAseq transcriptome anlaysis), showed higher expression in tegmental cells of miracidia and sporocysts. Within the tegument, relative expression (measured as counts per million of reads mapped) for genes of the hypothetical protein cluster ranged from 0 to a maximum of 1,496 and 6,236 in miracidia and sporocysts respectively while it only reached a maximum of 1.3 and 11 in adult males and females respectively (Supplementary Table 8). Consistent with whole organism (bulk) RNA-seq, limited expression was observed in the adult stages despite the presence of a tegument.

### A subtelomere-expanded cluster of Plexin Domain-Containing Genes show extensive sequence diversity and tegument-restricted expression

To investigate the extent of gene diversity within clusters, we compared gene model structures within each cluster (Supplementary Fig. 6). We observed that all clusters have extensive gene model variation, high level of fragmentation or are truncated. Analysis of the full complement of subtelomeric-expanded gene families is outside the scope of this work. However, to illustrate the complexity of gene structures found in this dynamic genomic region, we analysed the largest cluster of 49 “hypothetical proteins'' combining database searches and an -omics approach to illuminate potential protein function.

**Fig. 4:**
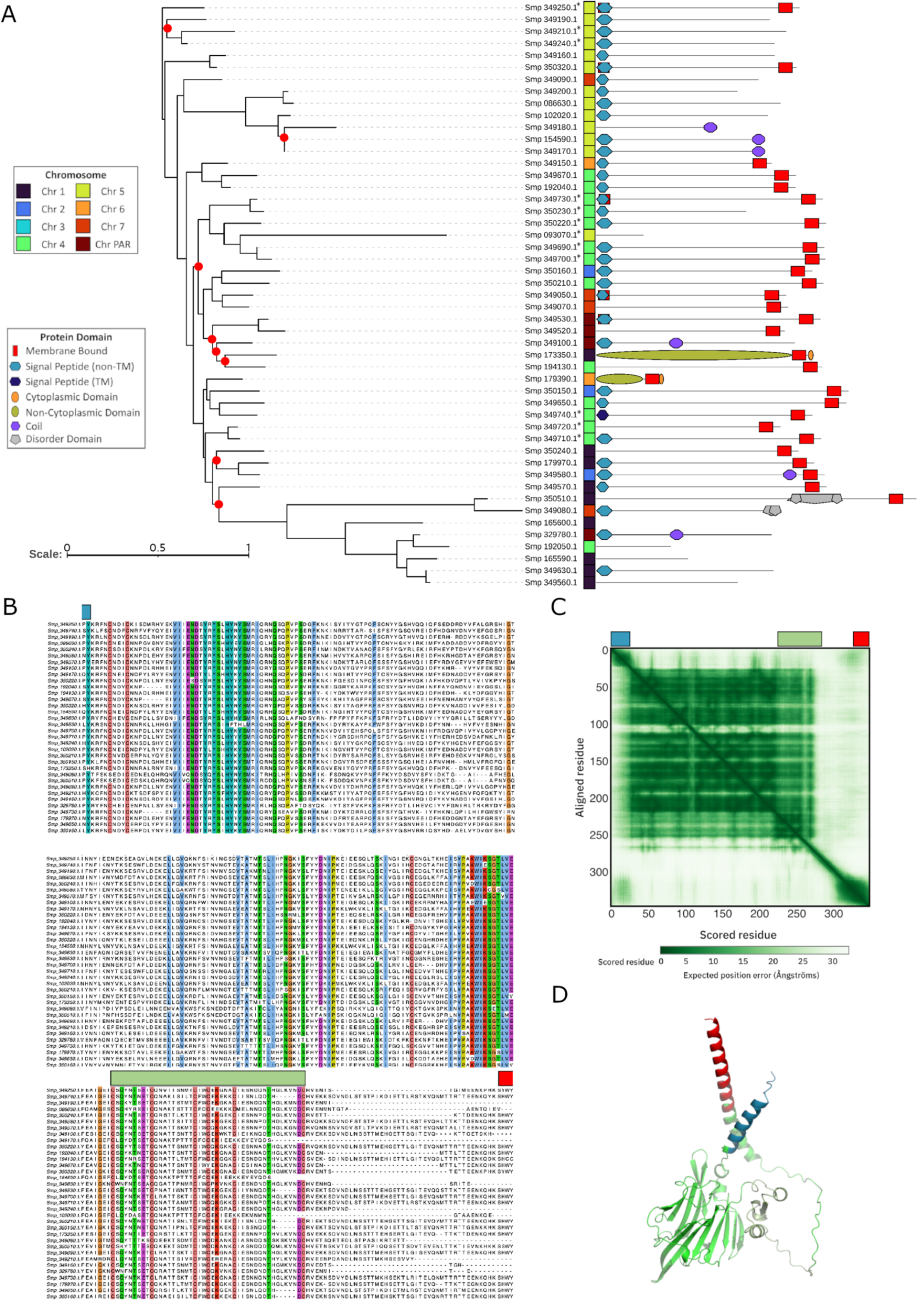
Proteins from the “hypothetical protein” cluster share a signal peptide, membrane-bound protein domain and significant similarity to plexin domain-containing proteins. **A**) Dendrogram of the amino acid alignment of proteins from the “hypothetical protein” cluster with corresponding protein domain annotation and chromosomal location to the right of figure. Asterisks next to gene identifier (Smp…*) denote non-subtelomeric gene copies. Red circles at nodes indicate an ultrafast bootstrap value < 70. Scale bar indicates number of nucleotide substitutions per codon. **B**) Multiple sequence alignment of selected hypothetical proteins where coloured residues indicate > 80% identity. Domains identified by InterProScan (blue and red) were trimmed for clarity, and the Plexin Semaphorin Integrin domain (green) inferred by hmmsearch was overlaid. **C**) Expected distance error (Ångströms) of one protein from the cluster (Smp_173350.1) using Alphafold with domains overlaid. **D**) Corresponding 3D protein model coloured according to the identified domains representative of this cluster

An exhaustive whole genome homology search revealed an additional 47 genes with similarity to members of this cluster (Supplementary Table 9). However, these do not take part in SDs and were therefore not included in further analyses. The segmentally duplicated genes belonging to this cluster have varied exon composition despite clustering by sequence identity and protein topology (Fig. 4A). We investigated the genomic location of each gene copy, finding copies in all chromosomes except Chromosome 3. Incongruence between sequence similarity and chromosome location can be seen across the dendrogram, highlighting the mobility of the gene cluster. For example, gene copies in Chromosome 4 do not cluster together, with the closest gene in sequence identity found on another chromosome in many cases (e.g. Smp_350150 and Smp_349650). Further accentuating this, of the genes’ non-subtelomeric copies, a fragment in Chromosome 5 clusters with genes of Chromosome 4 instead of the other copies outside the subtelomere of Chromosome 5.

Thirty-five of the 49 encoded proteins have an N-terminal signal peptide suggesting cell secretion/excretion, while 28 of these 35 also have a C-terminal membrane-bound domain suggesting cell surface retention. Protein domain searches using InterProScan [62] could not identify any significant matches for the approximately 300 amino acid internal section found between the signal peptide and the membrane-bound domain. However, multiple sequence alignment of these proteins suggested the presence of highly conserved residues (Fig. 4B) that formed a resolved structure (Fig. 4C). To explore the possibility of any as yet unannotated domains within this resolved, conserved protein core, we used the alignment to search for similar protein sequences using profile-HMMs [65]. Our search revealed significant similarity to a Plexin Sema Integrin (PSI) domain (Pfam accession PF01437) and various other proteins with “Plexin'' in their product description, despite no conserved domain annotation (E-value < 0.001, Supplementary Table 11), all of which are found outside the *Schistosoma* genus. These hits were present across distinct taxa, including 90 Actinopterdi (ray-finned fish) and 13 Insecta. Using the PSI’s (PF01437) seed sequences, we were able to identify the boundaries of this domain in our multiple sequence alignment of hypothetical protein (Supplementary File 7), confirming the presence of two pairs of conserved cysteines flanking a CxWC motif characteristic of plexin repeats (Fig. 4B, Green). Genes within the 49-copy segmentally duplicated gene cluster have historically been labelled “Egg protein CP-391S-like” and “Egg protein CP-391B-like” genes (Supplementary Table 12). Considering new evidence presented above, we rename these “plexin domain-containing protein”.

Finding a PSI domain in our newly named plexin domain-containing proteins only accounts for a fraction of the large and highly conserved amino acids block observed in the multiple sequence alignment (Fig. 4B.) To further explore this unknown region, we modelled the full protein's tertiary structure using Alphafold. At the time of writing, there was only one full-length entry (Smp_173350) of a *S. mansoni* plexin domain-containing protein cluster gene in the Alphafold database. We used this model to infer structural information regarding the wider cluster. The predicted aligned error plot (Fig. 4C) indicates the accuracy of the relative position of residues across the protein structure. The internal portion of this protein, between the signal peptide and membrane-bound domain, appears to form a single, high-confidence structure with well-resolved relative residue positions at the protein’s core. Whilst the PSI domain (green throughout Fig. 4) occupies a portion of the protein core, a larger but highly structured protein domain exists but we failed to find significant similarity to known protein domains in databases and remains uncharacterised (Fig. 4D) portion of the protein has no similarity with known protein domains and remains uncharacterised (Fig. 4D).

## Discussion

Research into subtelomeres has previously been difficult because they had been absent and when present, subtelomeres were highly fragmented and partially assembled, partially due to the region's intrinsic repetitiveness [1, 72]. This is particularly true for genomes of non-model organisms such as pathogens and parasites, which have been primarily assembled using short-read sequencing approaches and remain in a highly fragmented state [11]. As a result, the subtelomeres of many disease-causing organisms such as helminths are understudied and underappreciated despite their biological relevance. Technological advancements in genome sequencing are enabling the steady improvement of genome assemblies for many helminth species, including S. mansoni, the causative agent of the neglected tropical disease, schistosomiasis [17]. A near-telomere-to-telomere assembly leveraging long-read sequencing and HiC-assisted scaffolding is now available [24] and allows the analysis of these highly repetitive, poorly described regions. Our work characterises, for the first time, the location and plasticity of the S. mansoni subtelomere. These features are important in the genomic context because subtelomeres are regions that can undergo rapid evolution [4] and de novo gene formation [73, 74].

Even with a fully assembled genome, defining subtelomere boundaries is challenging, as different approaches to their classification exist [12, 75, 76] but none seemed applicable to the *S. mansoni* genome. To solve the subtelomere definition problem, we developed a novel method using an approach that captures the subtelomere’s intrinsic repetitiveness, which could be extrapolated upon a new assembly version of the same genome and may provide a non-species-specific subtelomere definition.

Repeat richness, relative to the rest of the genome, is a feature common to many organisms’ subtelomeres and is thought to stimulate non-allelic homologous recombination and SD formation [77] that, in turn, leads to gene density differences. The subtelomere’s intrinsic repetitiveness can therefore be used to define such regions and was chosen for the analysis of *S. mansoni* subtelomeres. Using manually curated repeat libraries that include TEs (Supplementary Table 1) and *S. mansoni*-specific W-elements [21], we observed increased repeat density at chromosome termini (Fig. 1A). We used a reproducible statistical approach of sliding 1 Mb windows and data-smoothing to identify significant repetitive element enrichment at these sites allowing us to define boundaries for *S. mansoni* subtelomeres (Fig. 1B & Table 1). With this approach we estimate that ~ 12% of the genome is subtelomeric.

The repetitive element enrichment that defines subtelomeres is TE-derived, as opposed to W-elements or tandem repeats (Fig. 1C), further reinforcing the need for manually curated repeat libraries, as automatic curation of TEs is often inaccurate [78]. We further demonstrate that *S. mansoni* subtelomeres are statistically gene-poor, with a median gene coverage of 0.469, compared the chromosome body’s mean coverage of 0.546 (Fig. 1C).

SDs, particularly at the subtelomere are sites of significant evolutionary relevance and fast evolving genes [4]. For example, the subtelomeres of the common bean (*Phaseolus vulgaris*) contains many resistance genes that have expanded via recombination [79]. SDs in *S. mansoni* have previously been explored [22] in a more fragmented, earlier genome assembly, likely omitting these highly relevant sites. In this work, the authors found that 0.22% of the genome was involved in SDs, encompassing 14 genes. Using an algorithm that designed to identified SDs and almost telomere-to-telomere genome assembly, we calculated that SDs occupy ~ 2.3% of the *S. mansoni* genome (Fig. 2A), representing a tenfold increase over previous reports. Moreover, we demonstrated that subtelomeric regions are significantly enriched in SD coverage (Fig. 2B) and that SDs occur at a disproportionately higher frequency at the subtelomere (Fig. 2C). Key differences in methodology between our work and that of Wang et al. (2017) include the use of a manually curated repeat library for the removal of repeat-element derived hits (an issue identified in their analysis) and our more stringent threshold applied to SD length, of 20 kb, as opposed to 10 kb [22].

However, our 2.3% calculation may still be an underestimate; some SDs may fall within the incomplete subtelomeres of the assembly (four out of the 16 chromosome ends lack telomeric repeats, Table 1) and there may be genuine SDs < 20 kb, which would be omitted from our dataset due to our length filtering. In addition, older SDs that might have once been > 20 kb may, over time, be interrupted by TEs and/or accumulated mutations such that they either fall beneath the length threshold or alignment quality filters and are therefore lost in our analysis. In fact, our results on nucleotide distance calculations for pairs of sequences forming SDs (Fig. 2D) show a range of evolutionary distances among SDs and may represent intermediate states between detectable and non-detectable duplications. Absence of zero values is evidence that none of these SDs are new, at least not in the reference genome here analysed.

The occurrence and stark distribution of SDs in the subtelomeres suggest that extensive non-allelic homologous recombination (NAHR) occurs between these regions. Such a phenomenon occurs due to repeat richness, which provides a homologous substrate from which NAHR can happen [80, 81]. It is, therefore, not surprising that most subtelomeric rearrangements in other organisms are NAHR-mediated [82] and that *S. mansoni*’s repeat-rich subtelomeres may be substrate for NAHR, resulting in significant SD enrichment in all but two ends of two chromosomes (chr 1 and 3, Fig. 2A). We conclude that repeat richness is important in SD formation in *S. mansoni*, with this notion being backed up by examples in other organisms. However, our study of repeat content flanking SD breakpoints (Supplementary Fig. 1) did not reveal any patterns suggesting involvement of a specific repeat type in SDs formation.

It seems likely that the intrinsic distance and proximity between subtelomere and chromosome end is also a contributor to recombination dynamics. This is suggested by two observations made in our analyses: i) non-subtelomeric repeat-rich regions (Fig. 1B) do not participate in rearrangements or the expansion of gene clusters and ii) whilst a wide distribution of genes is observed around subtelomeres, those resulting from SDs were often some of the closest to the chromosome end (Supplementary Fig. 3). A potential hypothesis described in P. falciparum var gene expansion [83] is that upon bouquet formation, a stage of meiosis in which telomeres adhere to the nuclear membrane, chromosome ends are in close physical proximity to one another [84], stimulating recombination. In humans, most SDs occur between chromosome termini, much the same as in other organisms; however, this phenomenon is restricted to the short arms of acrocentric chromosomes [9]. This chromosomal polarity is not mirrored in *S. mansoni*, as SDs occur at both ends of five of the eight chromosomes (Fig. 1A). It seems likely that a combination of repeat richness, which stimulates NAHR and SD formation, and the physical characteristics of the chromosome termini are required for the dynamics observed at the *S. mansoni* subtelomere. Though in this analysis, we were unable to provide further mechanistic insight into the inter-chromosomal exchange described. Visualisation of chromosomal and subtelomeric interactions during mitosis and meiosis, especially incorporating comparative studies between subtelomeres of different organisms, may be useful to understand the driving forces behind subtelomeric SD formation observed in *S. mansoni*.

Gene family expansion is an evolutionarily important mechanism for increasing gene dosage, removing evolutionary constraints, and allowing functional diversification [85] and has long been associated with subtelomeres in many organisms [12–15]. We used a gene-independent approach to identify inter-chromosomal SDs by requiring non-repeat derived homology greater than 20 kb in length between two sites. This allows mapping of bona fide recombination events instead of homology deriving from, for example, conserved protein domains or misannotated TEs. Our results showed that SDs in *S. mansoni* subtelomeres have resulted in the expansion and duplication of 103 genes that cluster (by sequence homology) into nine discrete families (Fig. 3A). These gene families expanded via SDs include the annexin family, which are involved in host immune response modulation [86], maintain membrane and tegument integrity [87], and are considered putative drug or vaccine targets [88]. This somehow mirrors the arrangement of subtelomeric genes of two other medically important parasites P. falciparum and Trypanosoma spp., in which the var and vsg subtelomeric gene families respectively, facilitate host immune system evasion by antigenic variation [89, 90].

A potential benefit and outcome from gene duplication is sub-functionalisation or neo-functionalisation, in which duplicated genes acquire new functions or are turned on at different stages of development [91]. We hypothesise that this could be the case for the annexin cluster, where two groups of genes can be distinguished by their expression in either the snail or the mammalian host (Fig. 3B), suggesting at least temporal compartmentalisation of gene function. Perhaps, in this case, duplication has allowed for independent mutation of the separate groups and the generation of specificity towards the two different hosts and diverse expression patterns. Despite life-stage specificity, just four genes showed upregulated expression in given cell types and were consistently more highly expressed in the parenchyma. Other subtelomeric clusters include “Endoglycoceramidases” (*n* = 14) and “Conserved Oligomeric Golgi Complex Subunit 4” (*n* = 8). Little is known about these clusters and, given the relevance of subtelomeres in other genomes [92], the multicopy nature of the loci (Fig. 3A) and the stage-specific expression observed (Fig. 3B) warrants further study. In addition to the nine gene clusters expanded around the subtelomere, our results include at least two examples of subtelomere-independent expansions that occur due to tandem duplication (Supplementary Fig. 2). Whilst these tandem duplications further demonstrate the evolutionary benefit of gene expansion, they do not represent NAHR and fall outside the scope of this work (Cercarial Proteases and Mucins, Fig. 3A).

The largest example of SD gene family expansion is a cluster of 49 genes, of which 36 were subtelomeric (Fig. 4A), of unknown function but with some genes annotated as “CP-391B-like egg proteins” and “CP-391S-like egg proteins”. Using a combination of database searches and in silico structural analyses we found that a highly conserved region of these proteins is significantly similar to a plexin domain-containing protein, based on the identification of a conserved plexin-repeat domain with a characteristic “CxWC” motif (Fig. 4B) [93, 94]. We could not identify any protein domains associated with the remaining part of the protein core despite high conservation throughout the predicted polypeptide (Fig. 4B) and a well-resolved core structure upstream of the plexin domain (Figs. 4C and 4D). Little is known about PSI domains, particularly their direct biochemical function [95]; however, they are often subunits of larger, functional often extracellular receptor domains [93] where they have at least a structural role in signal transduction [96]. We hypothesise that the remaining unannotated protein core represents a larger domain, within which the PSI domain is a subunit. Similar protein domain architecture is found in platyhelminth-specific integrins, which have a 5’ signal peptide, a 3’ membrane-bound domain and core integrin domains, of which PSI is a subunit [97]. Because subtelomeric genes are often taxa-specific owing to the rapidly changing interface at which they are found, using databases mainly informed by model organisms limits our capacity to identify parasite-specific protein domains [71, 98, 99]. Similar proteins are, therefore, unlikely to be present in orthogonal datasets from which functional and structural inferences can be drawn. This is likely to change soon due to the increasing number of publicly available genomes.

We combined whole organism and single cell transcriptome data for various life cycle stages of the parasite and found that plexin domain-containing proteins are exclusively overexpressed in the tegument of the intra-molluscan stages of the parasite (Fig. 3B). This hints at a specific functional role within the intramolluscan stage, as opposed to a more constitutive, structural tegument protein. The tegument, being the schistosomes’ outer layer, is the primary site of host-parasite interaction [100] and, as such, is the logical site for therapeutic targets of vaccines [101]. Other Schistosoma tegument gene families, such as tetraspanins, have long been of interest due to their role in host’s immune modulation [101, 102] and are potential therapeutic targets [103].

Expansion of subtelomeric gene families is a highly important process leading to increased virulence and survival in unicellular parasites. Here, we explicitly define and characterise the subtelomere and a similar phenomenon occurring in a metazoan parasite. Evidence of subtelomere-associated *hsp70* expansion in tapeworms [104], a gene potentially also acting at the host-parasite interface in secreted extracellular vesicles [105], presents the metazoan parasite subtelomere as a region of interest. For the first time, we identify the *S. mansoni* subtelomeres, demonstrate their highly plastic nature and explore the potential implications for gene family expansion occurring by subtelomeric segmental duplications. This includes the identification of 49 segmentally duplicated copies of a plexin domain-containing protein gene cluster. This appropriately demonstrates the value of subtelomere analysis and further work may shed light on the yet unknown mechanisms of host immune evasion that schistosomes employ to survive in their hosts undetected for decades [106].

## Conclusion

This work describes *S. mansoni*’s subtelomeres as a hotspot of non-allelic homologous recombination in which segmental duplications (SDs) and gene family expansion occur disproportionately when compared to the rest of the genome. Our work identified 10 times more SDs than previously reported and expanded the number and nature of their gene content demonstrating the benefit of improved assemblies and gene finding when dealing with complex, large-scale repetitive regions such as subtelomeres. Given that subtelomeric SDs and the potentially relevant gene families identified at these sites were entirely undescribed before the improved *S. mansoni* assembly, other high-quality parasite genomes, including additional *Schistosoma spp.* would benefit from similar analyses. Comparative analysis between high-quality genomes may allow for the identification of mechanisms driving subtelomeric recombination and may uncover gene families relevant for parasite survival and host-parasite interactions.

### Supplementary Information

**Additional file 1: Supplementary Figure 1.**

**Additional file 2: Supplementary Figure 2. **

**Additional file 3: Supplementary Figure 3. **

**Additional file 4: Supplementary Figure 4. **

**Additional file 5: Supplementary Figure 5. **

**Additional file 6: Supplementary Figure 6. **

**Additional file 7: Supplementary file 1. **

**Additional file 8: Supplementary file 2. **

**Additional file 9: Supplementary file 3. **

**Additional file 10: Supplementary file 4. **

**Additional file 11: Supplementary file 5. **

**Additional file 12: Supplementary file 6. **

**Additional file 13: Supplementary file 7.**

**Additional file 14: Supplementary Table 1. **

**Additional file 15: Supplementary Table 2. **

**Additional file 16: Supplementary Table 3. **

**Additional file 17: Supplementary Table 4. **

**Additional file 18: Supplementary Table 5. **

**Additional file 19: Supplementary Table 6. **

**Additional file 20: Supplementary Table 7. **

**Additional file 21: Supplementary Table 8. **

**Additional file 22: Supplementary Table 9. **

**Additional file 23: Supplementary Table 10. **

**Additional file 24: Supplementary Table 11. **

**Additional file 25: Supplementary Table 12. **

## Data Availability

Code used in analysis is available on Github at: https://github.com/tbrann99/Subtelomere Supplementary files can be found in native formats at: https://zenodo.org/records/10143033
