# Supplementary material for: Subtelomeric plasticity contributes to gene family expansion in the human parasitic flatworm Schistosoma mansoni
Source: BMC Genomics. 2024 Feb 27;25:217. doi: 10.1186/s12864-024-10032-8 (PMC10900676; doi:10.1186/s12864-024-10032-8)
Supplement: Supplementary file 1 — Additional file 1: Supplementary Figure 1. [file 12864_2024_10032_MOESM1_ESM.pdf]

A

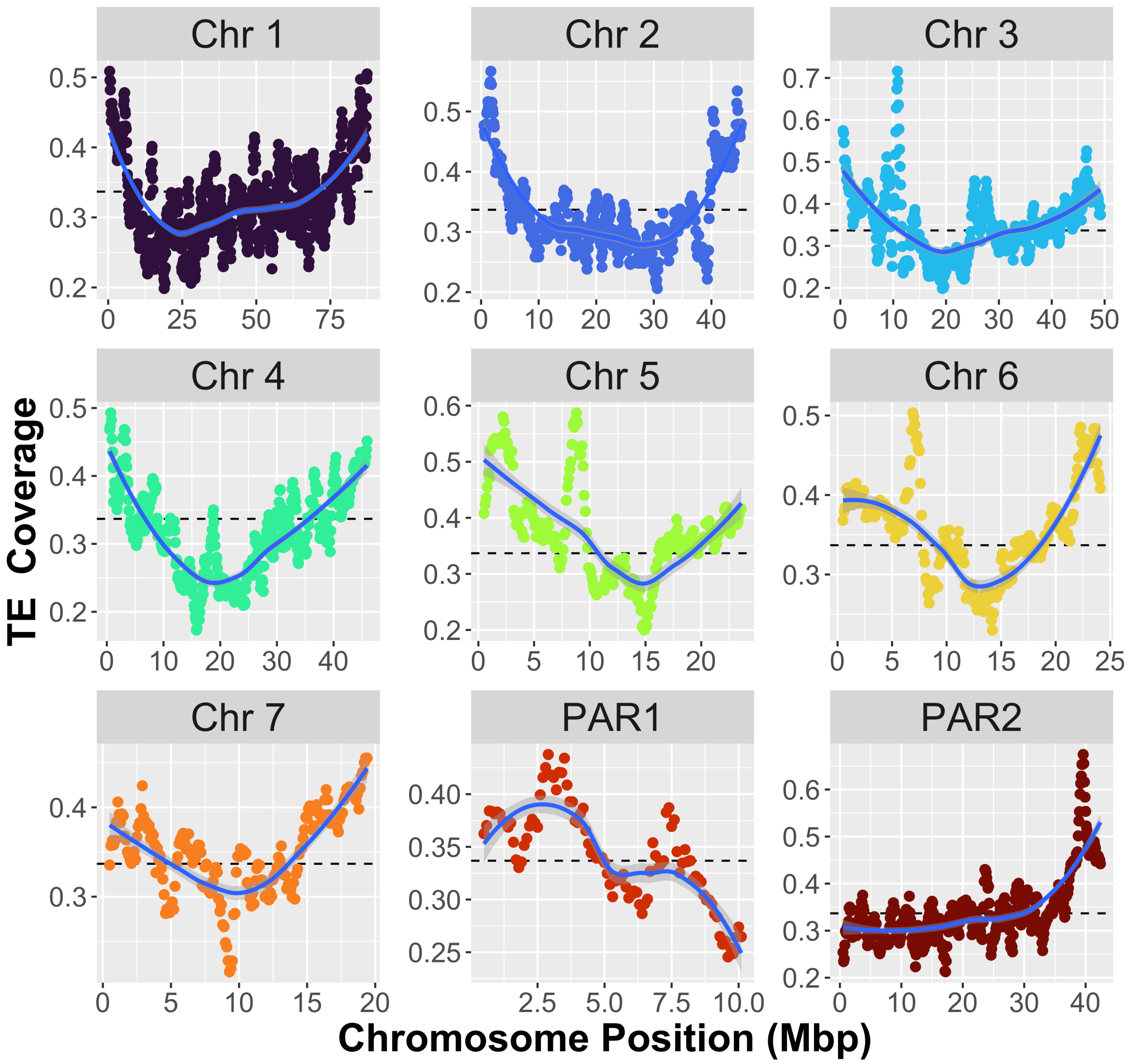

Chr 1
Chr 2
Chr 3
Chr 4
Chr 5
Chr 6
Chr 7
PAR1
PAR2
TE Coverage
Chromosome Position (Mbp)

B

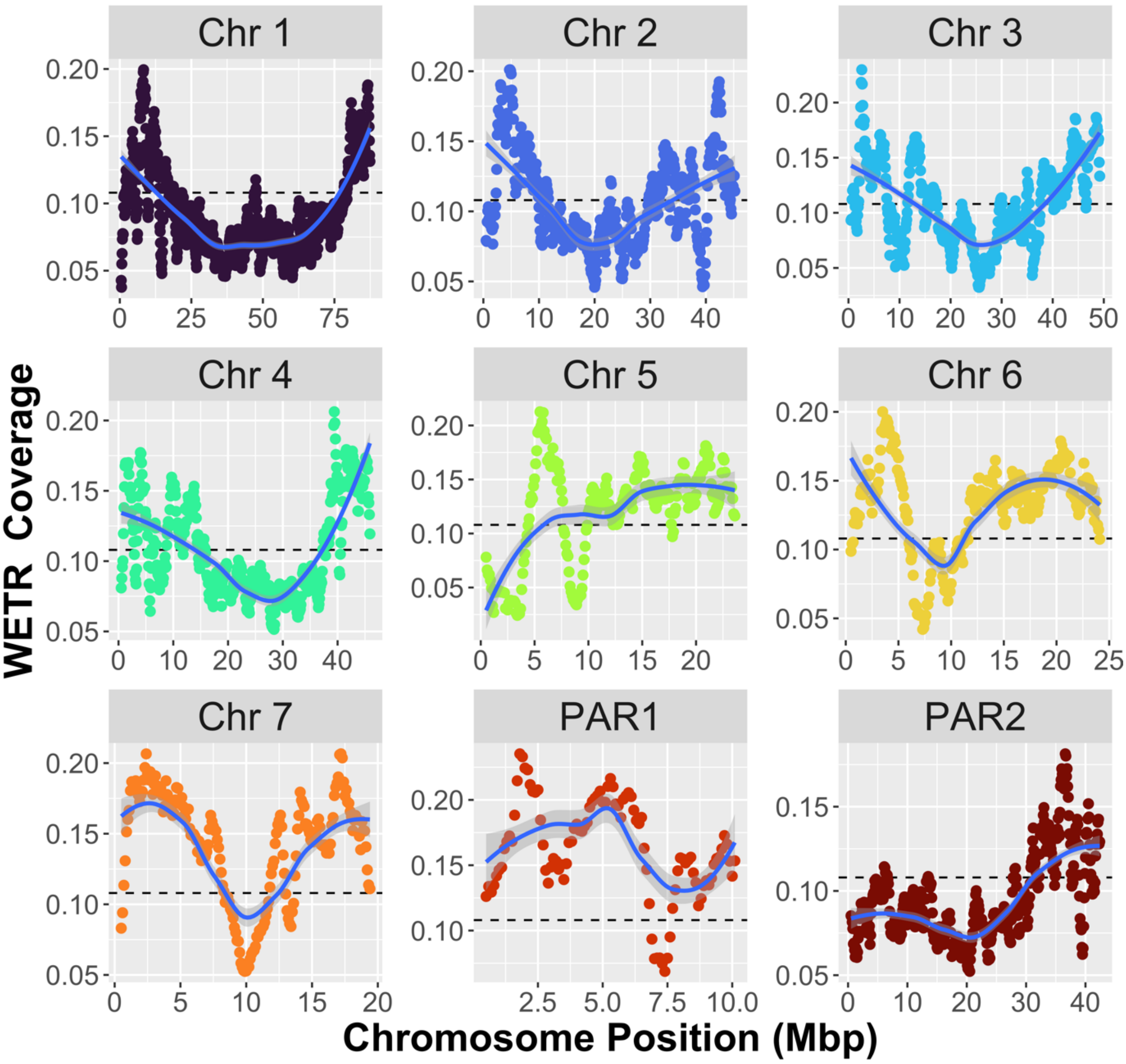

Chr 1
Chr 2
Chr 3
Chr 4
Chr 5
Chr 6
Chr 7
PAR1
PAR2
WETR Coverage
Chromosome Position (Mbp)

C

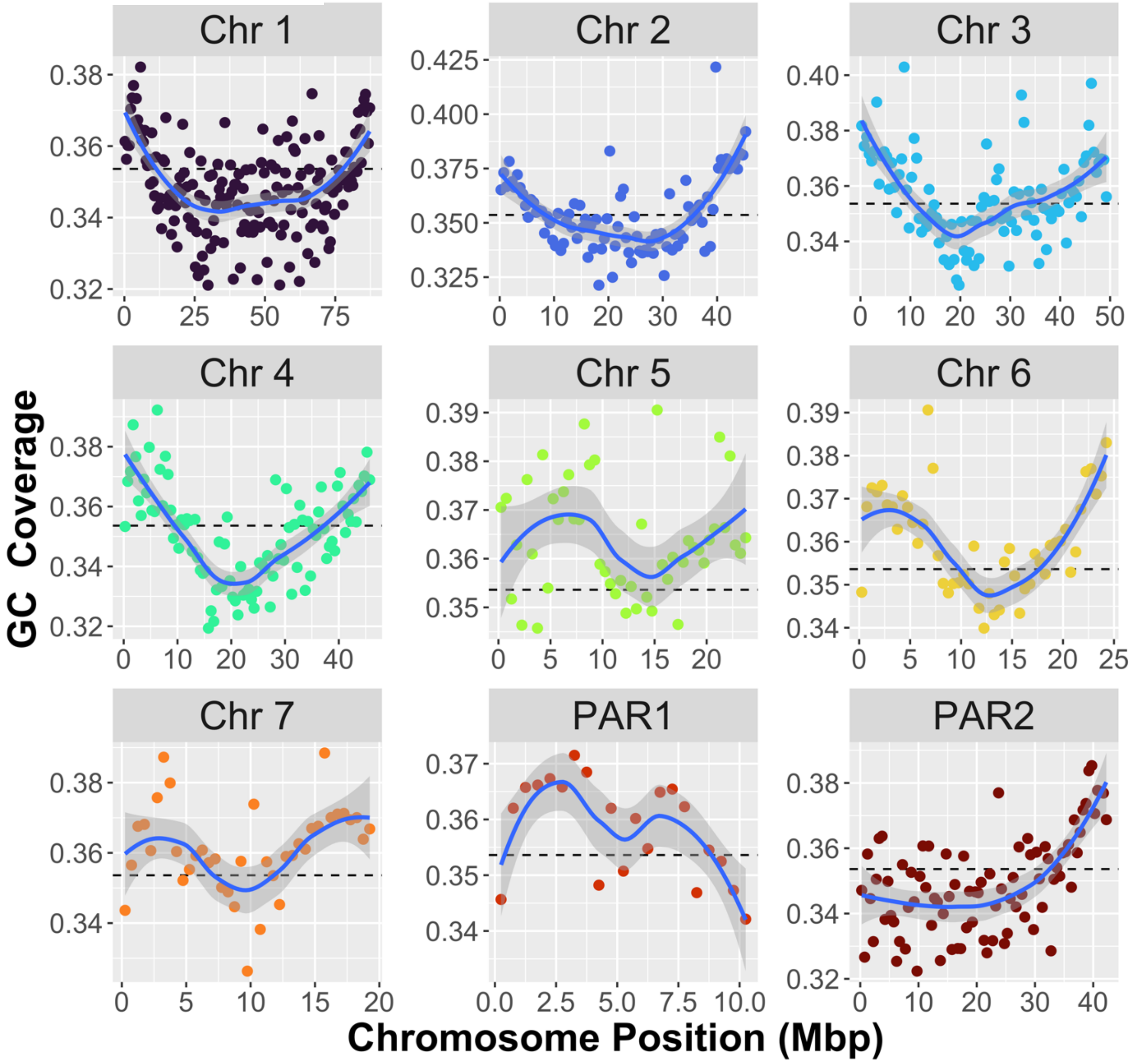

Supplementary Figure 1 – Coverage plots of (A) Transposable Elements (TEs), (B) W-Elements and tandem repeats (WETR) and (C) GC content across *S. mansoni* chromosomes. All data points plotted in the same windows as Figure 1 (1Mbp windows with 100kbp steps). Black dashed line indicates genomic mean and curved blue line implemented with the geom_smooth function to demonstrate trends across chromosomes.
