# Supplementary material for: Subtelomeric plasticity contributes to gene family expansion in the human parasitic flatworm Schistosoma mansoni
Source: BMC Genomics. 2024 Feb 27;25:217. doi: 10.1186/s12864-024-10032-8 (PMC10900676; doi:10.1186/s12864-024-10032-8)
Supplement: Supplementary file 2 — Additional file 2: Supplementary Figure 2. [file 12864_2024_10032_MOESM2_ESM.pdf]

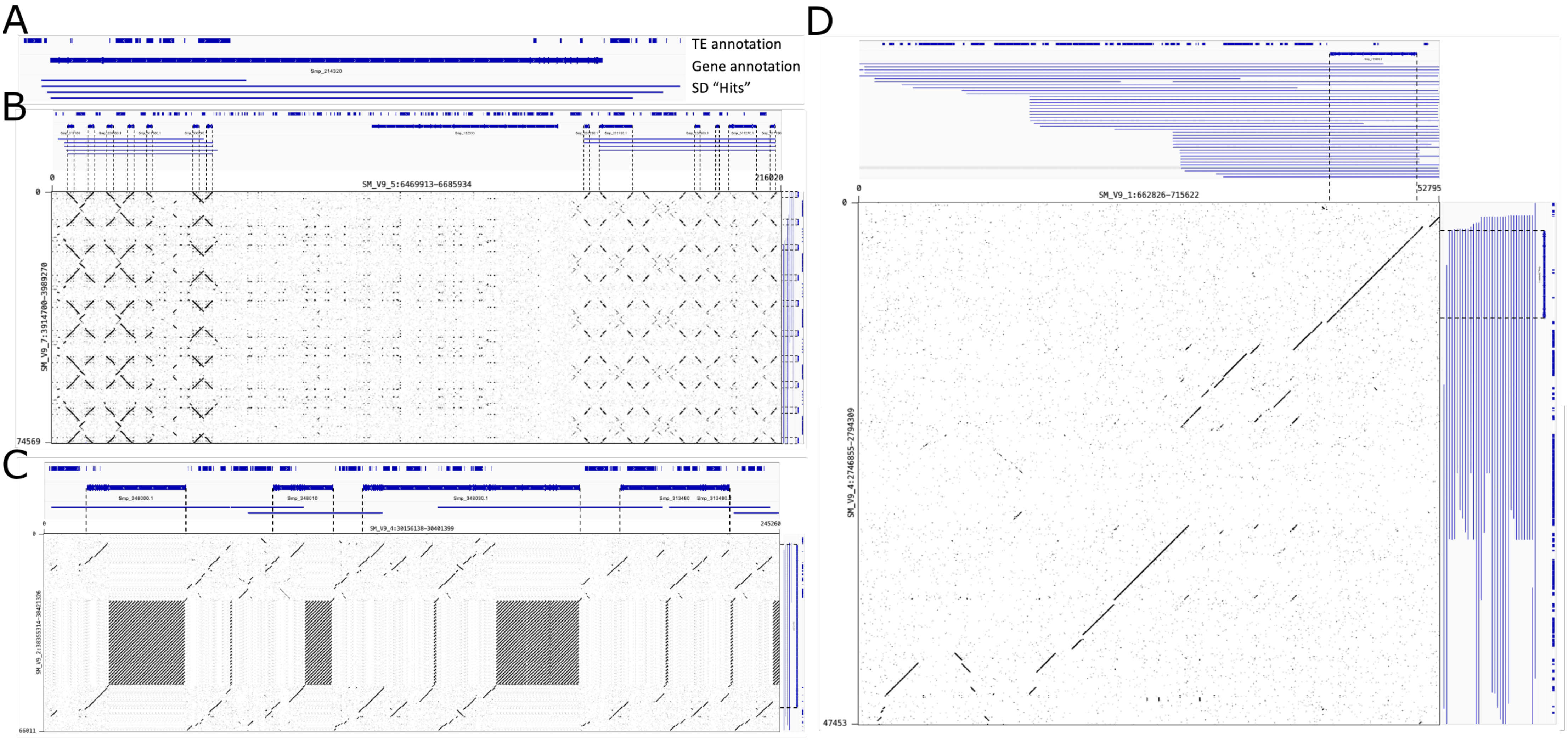
A
TE annotation
Gene annotation
SD "Hits"
Smp_214320
B
SM_V9_5:6469913-6685934
0
216020
SM_V9_7:3914700-3989270
74569
C
Smp_348000.1
Smp_348010
Smp_348030.1
Smp_313480
SM_V9_4:30156138-30401399
245260
SM_V9_2:38355314-38421326
66011
D
SM_V9_1:662826-715622
52795
SM_V9_4:2746855-2794309
47453

Supplementary Figure 2 – Demonstration of tandem duplicated hits in dataset. A - Key for reading IGV tracks for the figure. Tracks include transposable element (TE) annotations, the gene annotations from the V10 pre-released (V9.5 - SM_V9_updated_221114.gff3) and “SD_hits.bed”, the bedfile of biser’s filtered output. Black dashed lines are overlaid to indicate the position of genes more clearly on the dotplot. **B** - Segmental duplications across the two cercarial protease loci of chromosome 5 (which are approx 200kbp away from one another) and chromosome 7. Note the lack of continuity from chromosome 5 to chromosome 7, indicating the genes have been tandemly duplicated independently of the other loci, as we observe no continued homology throughout the region. **C** - Segmental duplications across the four mucins of chromosome 4 and the mucin on chromosome 2. Gene models have been duplicated around the large (unannotated) tandem repeats present at the loci, as can be seen in the similarity between the 5’ and 3’ and of the same mucin genes. **D** - Example of segmental duplication with two of the hypothetical proteins, in which near unidirectional homology is observed across a larger region than the gene itself.
