# Supplementary material for: Subtelomeric plasticity contributes to gene family expansion in the human parasitic flatworm Schistosoma mansoni
Source: BMC Genomics. 2024 Feb 27;25:217. doi: 10.1186/s12864-024-10032-8 (PMC10900676; doi:10.1186/s12864-024-10032-8)
Supplement: Supplementary file 3 — Additional file 3: Supplementary Figure 3. [file 12864_2024_10032_MOESM3_ESM.pdf]

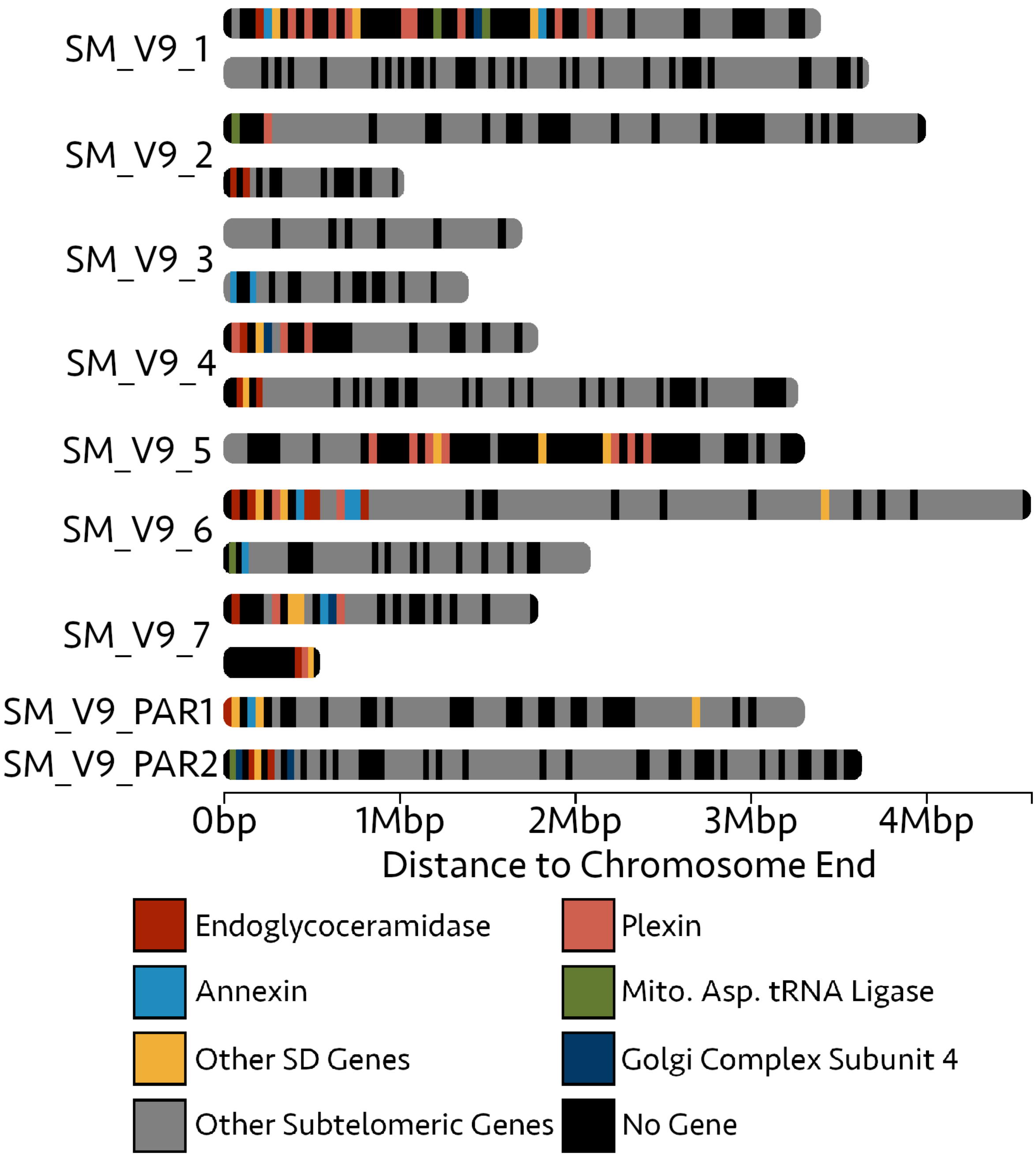

Supplementary Figure 3 – Chromomap of Segmentally Duplicated Gene Clusters around *Schistosoma mansoni* subtelomeres. For chromosomes except SM_V9_5, PAR1 and PAR2, two chromosome ends are shown per chromosome. In all cases, the top segment, indicates the chromosome end that begins at position "1bp" on each chromosome and the bottom, the opposite. Irrespective of chromosome end (5' or 3'), the left side indicates the position closest to the chromosome end. Genes were annotated in accordance with the SSN, with exception of two-gene and single gene clusters which were reclassified to "Other SD Genes". "Other Subtelomeric Genes" refers to subtelomeric genes that do not participate in segmental duplications. "No Gene" refers to the chromosomal region, with no annotated genes. Though positions are to scale, there is a fixed width to displaying annotations on the chromosome, which may make genes appear closer to one another or closer to the chromosome end than in practice.
