# Supplementary material for: Subtelomeric plasticity contributes to gene family expansion in the human parasitic flatworm Schistosoma mansoni
Source: BMC Genomics. 2024 Feb 27;25:217. doi: 10.1186/s12864-024-10032-8 (PMC10900676; doi:10.1186/s12864-024-10032-8)
Supplement: Supplementary file 4 — Additional file 4: Supplementary Figure 4. [file 12864_2024_10032_MOESM4_ESM.pdf]

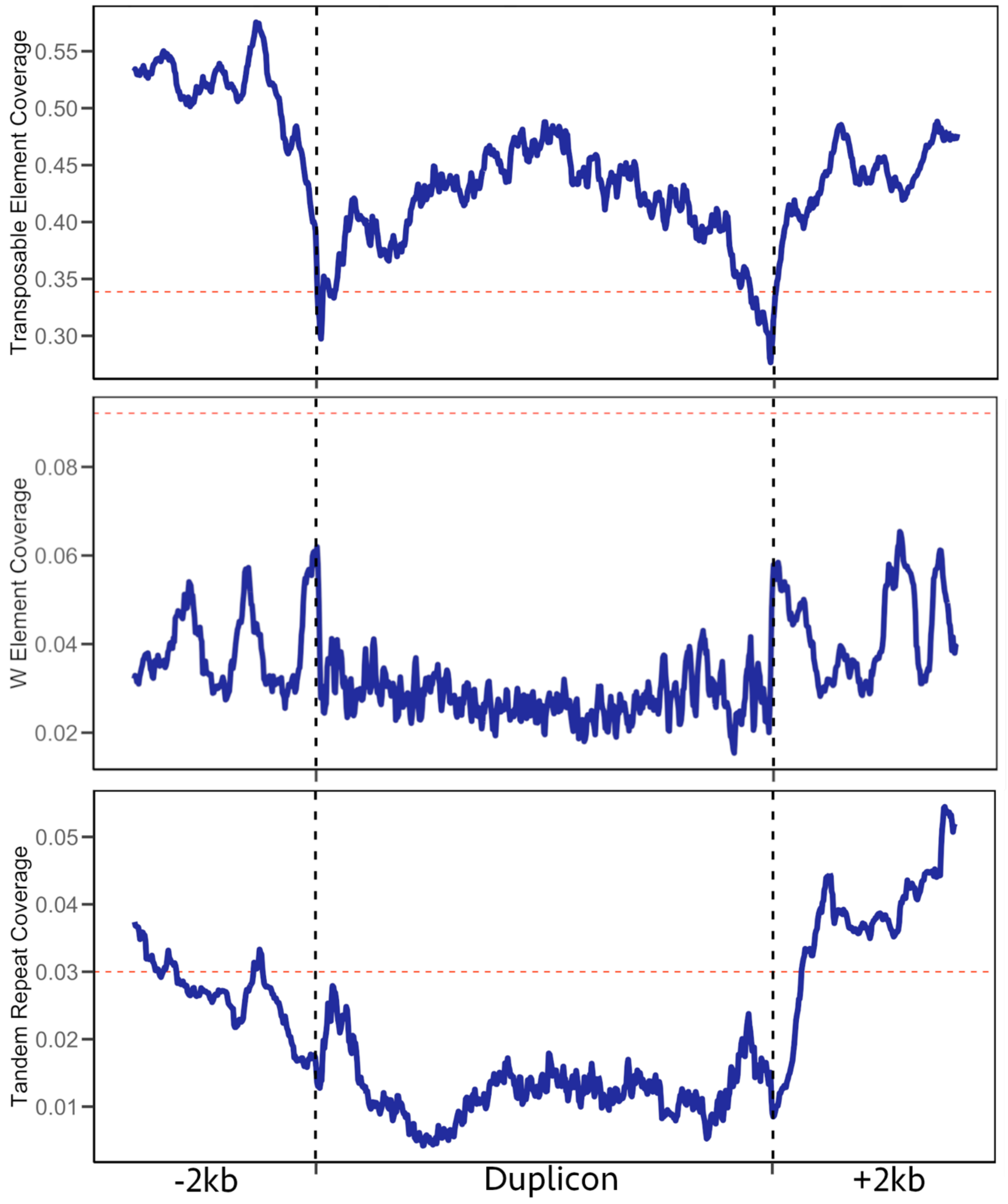

Supplementary Figure 4 – Coverage of segmental duplications (duplicons) with repeat elements (transposable elements, W-Elements and tandem repeats). Window for each respective duplicon extended 2,000bp upstream and downstream. Red dashed line indicates the genomic mean for the respective repetitive element.
