# Supplementary material for: Subtelomeric plasticity contributes to gene family expansion in the human parasitic flatworm Schistosoma mansoni
Source: BMC Genomics. 2024 Feb 27;25:217. doi: 10.1186/s12864-024-10032-8 (PMC10900676; doi:10.1186/s12864-024-10032-8)
Supplement: Supplementary file 5 — Additional file 5: Supplementary Figure 5. [file 12864_2024_10032_MOESM5_ESM.pdf]

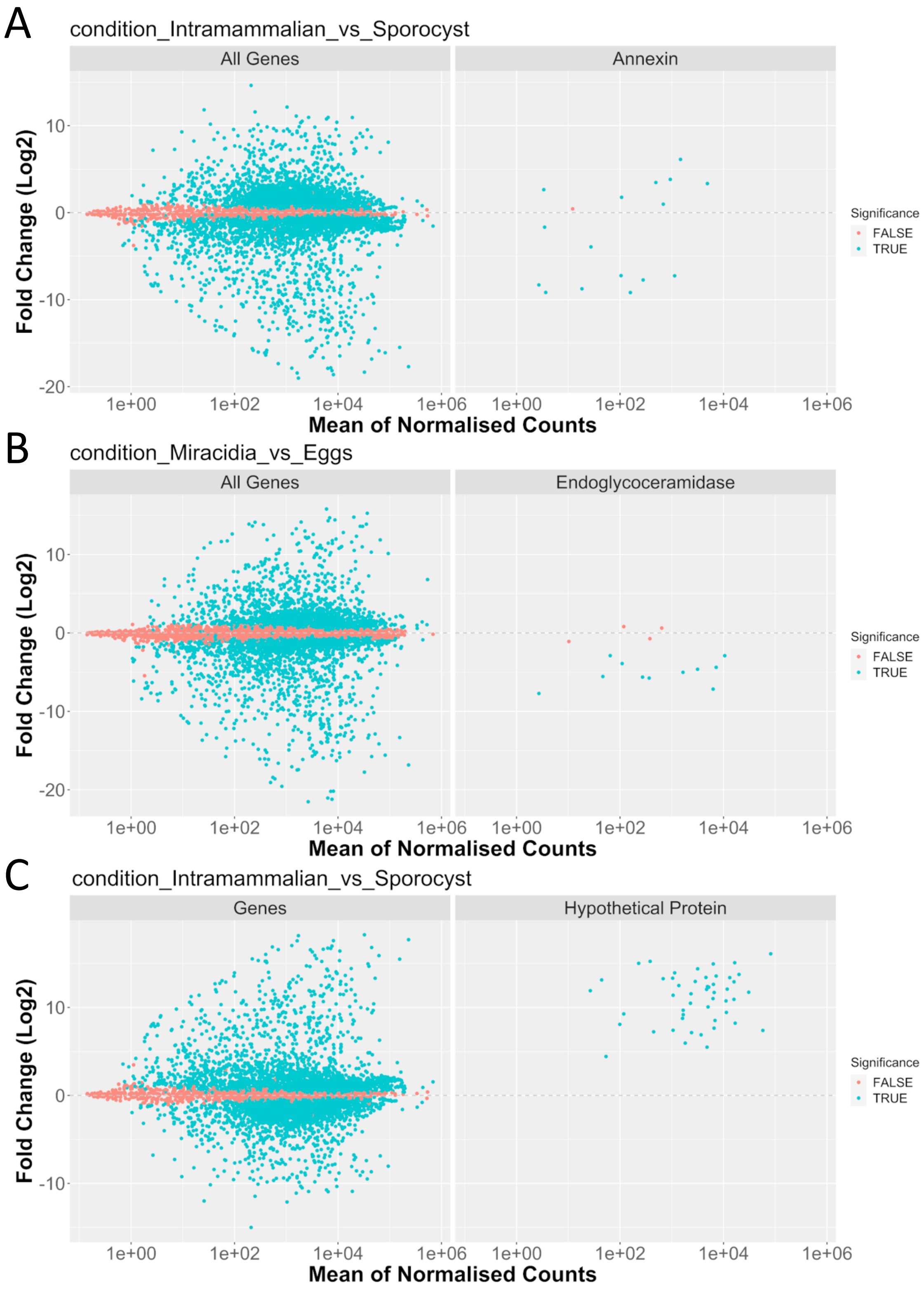

Supplementary Figure 5 – MA plots of relevant timepoints and subtelomerically expanded gene clusters. (A) – Log fold change of Annexin gene expression between intramammalian and sporocyst life stages, **(B)** – Log fold change of endoglycoceramidases between miracidia and egg life stages, **(C)** – Log fold change of hypothetical protein cluster between intrammammalian and sporocyst life stages. Intramammalian refers to the mean of schistosomula and juvenile stages samples and sporocyst the 1, 5 and 32 day samples. Blue indicates genes which were significantly differentially expressed between stages ($p_{adj} < 0.05$).
