# Supplementary material for: Subtelomeric plasticity contributes to gene family expansion in the human parasitic flatworm Schistosoma mansoni
Source: BMC Genomics. 2024 Feb 27;25:217. doi: 10.1186/s12864-024-10032-8 (PMC10900676; doi:10.1186/s12864-024-10032-8)
Supplement: Supplementary file 6 — Additional file 6: Supplementary Figure 6. [file 12864_2024_10032_MOESM6_ESM.pdf]

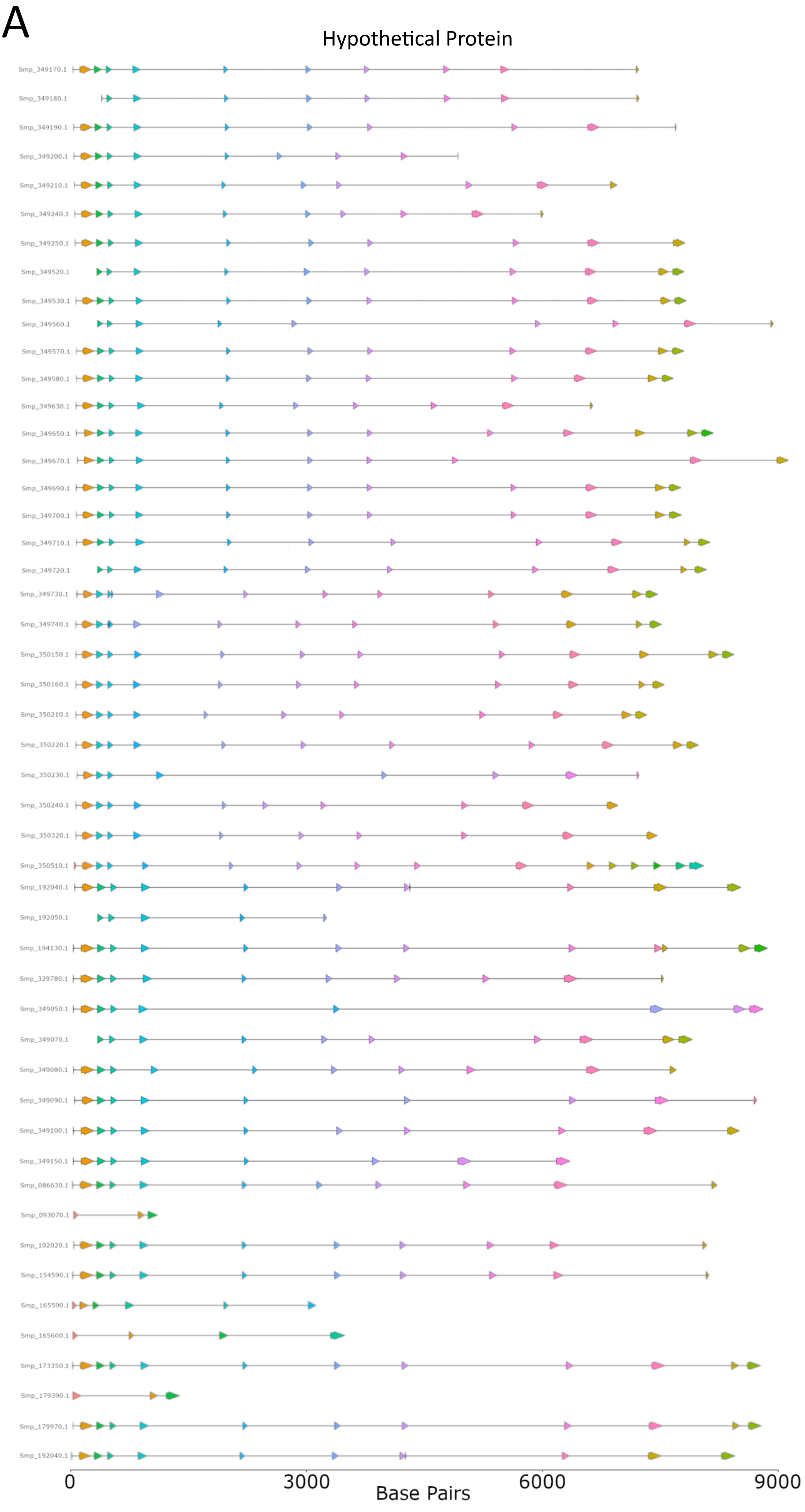
A
Hypothetical Protein
Smp_349170.1
Smp_349180.1
Smp_349190.1
Smp_349200.1
Smp_349210.1
Smp_349240.1
Smp_349250.1
Smp_349520.1
Smp_349530.1
Smp_349560.1
Smp_349570.1
Smp_349580.1
Smp_349630.1
Smp_349650.1
Smp_349670.1
Smp_349690.1
Smp_349700.1
Smp_349710.1
Smp_349720.1
Smp_349730.1
Smp_349740.1
Smp_350150.1
Smp_350160.1
Smp_350210.1
Smp_350220.1
Smp_350230.1
Smp_350240.1
Smp_350320.1
Smp_350510.1
Smp_192040.1
Smp_192050.1
Smp_194130.1
Smp_329780.1
Smp_349050.1
Smp_349070.1
Smp_349080.1
Smp_349090.1
Smp_349100.1
Smp_349150.1
Smp_086630.1
Smp_093070.1
Smp_102020.1
Smp_154590.1
Smp_165590.1
Smp_165600.1
Smp_173350.1
Smp_179390.1
Smp_179970.1
Smp_192040.1
0
3000
6000
9000
Base Pairs

B

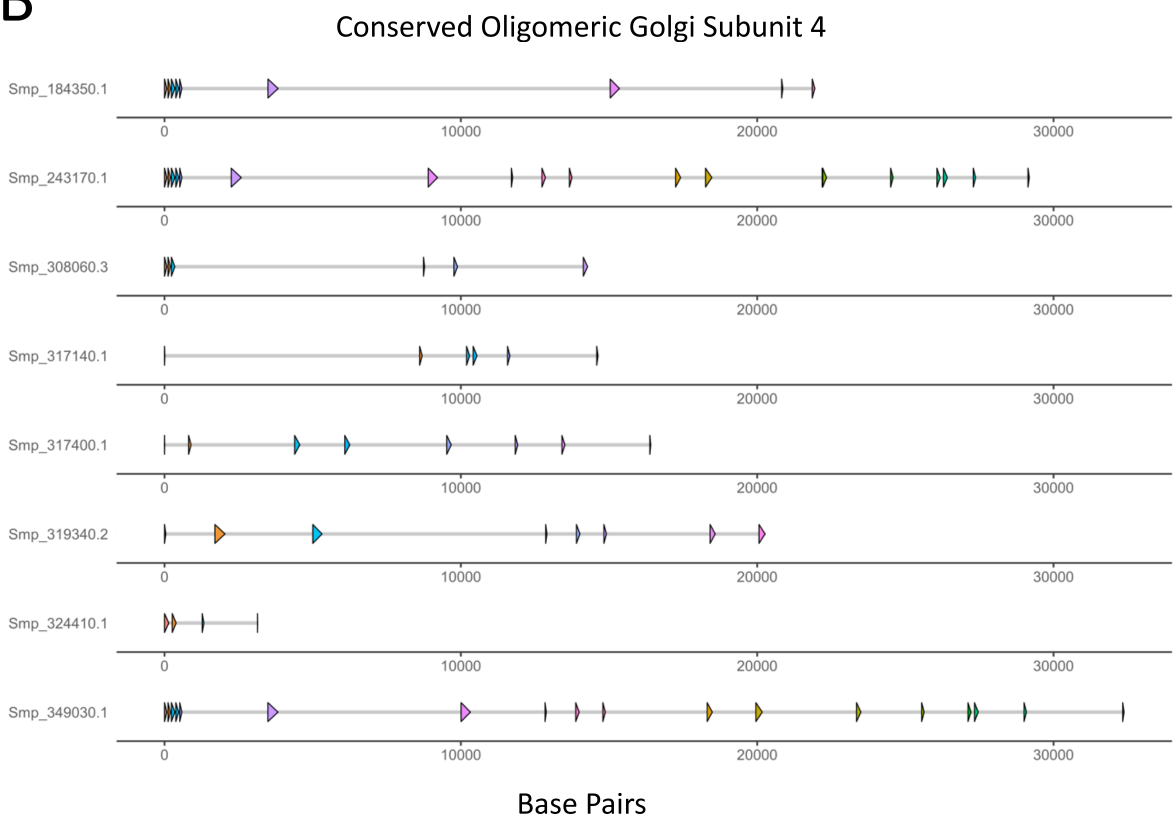

Conserved Oligomeric Golgi Subunit 4
Smp_184350.1
Smp_243170.1
Smp_308060.3
Smp_317140.1
Smp_317400.1
Smp_319340.2
Smp_324410.1
Smp_349030.1
0
10000
20000
30000
Base Pairs

C

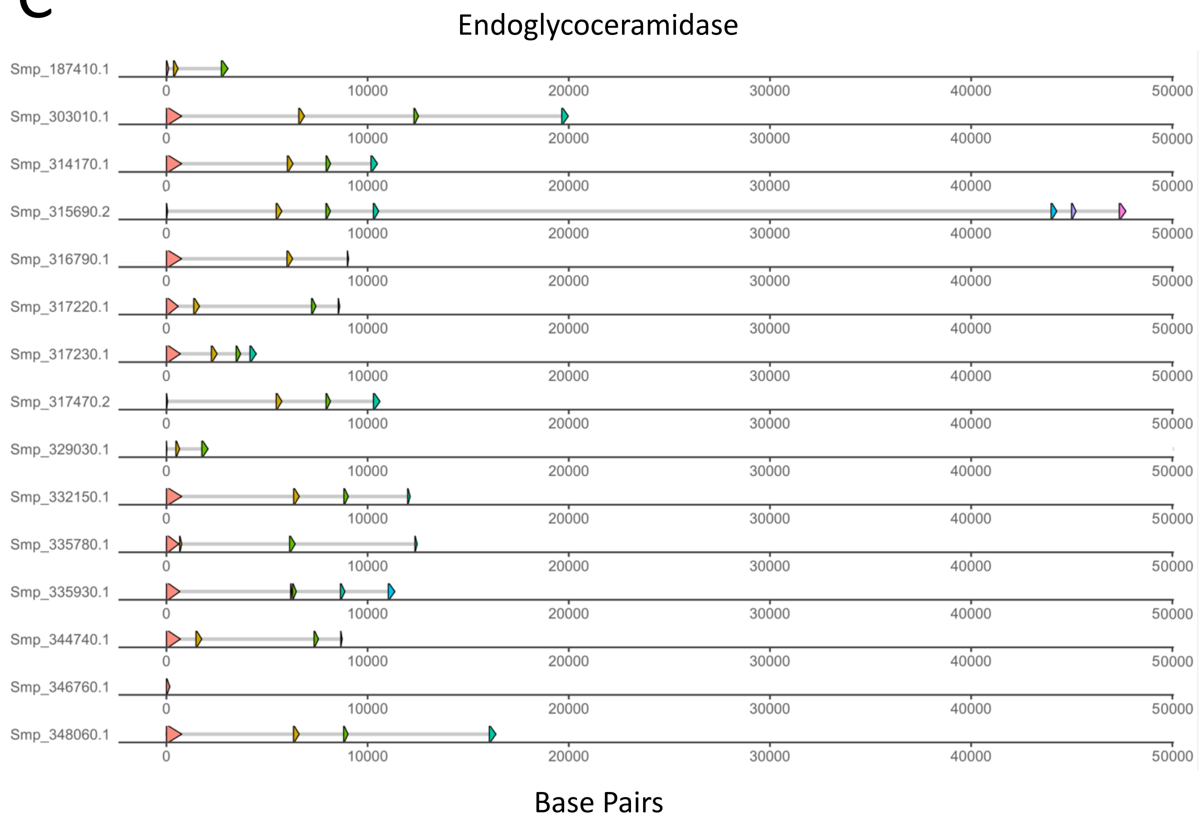
Endoglycoceramidase
Smp_187410.1
Smp_303010.1
Smp_314170.1
Smp_315690.2
Smp_316790.1
Smp_317220.1
Smp_317230.1
Smp_317470.2
Smp_329030.1
Smp_332150.1
Smp_335780.1
Smp_335930.1
Smp_344740.1
Smp_346760.1
Smp_348060.1
0
10000
20000
30000
40000
50000
Base Pairs

D

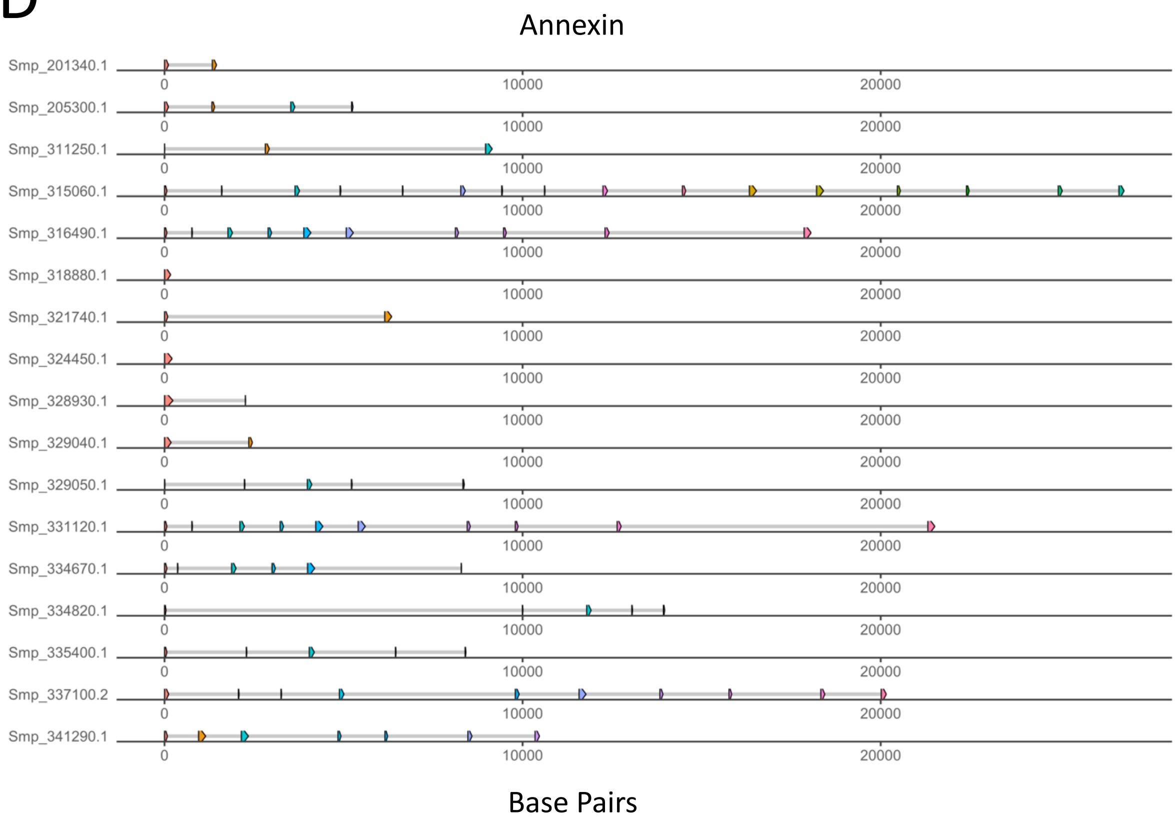
Annexin
Smp_201340.1
Smp_205300.1
Smp_311250.1
Smp_315060.1
Smp_316490.1
Smp_318880.1
Smp_321740.1
Smp_324450.1
Smp_328930.1
Smp_329040.1
Smp_329050.1
Smp_331120.1
Smp_334670.1
Smp_334820.1
Smp_335400.1
Smp_337100.2
Smp_341290.1
0
10000
20000
Base Pairs

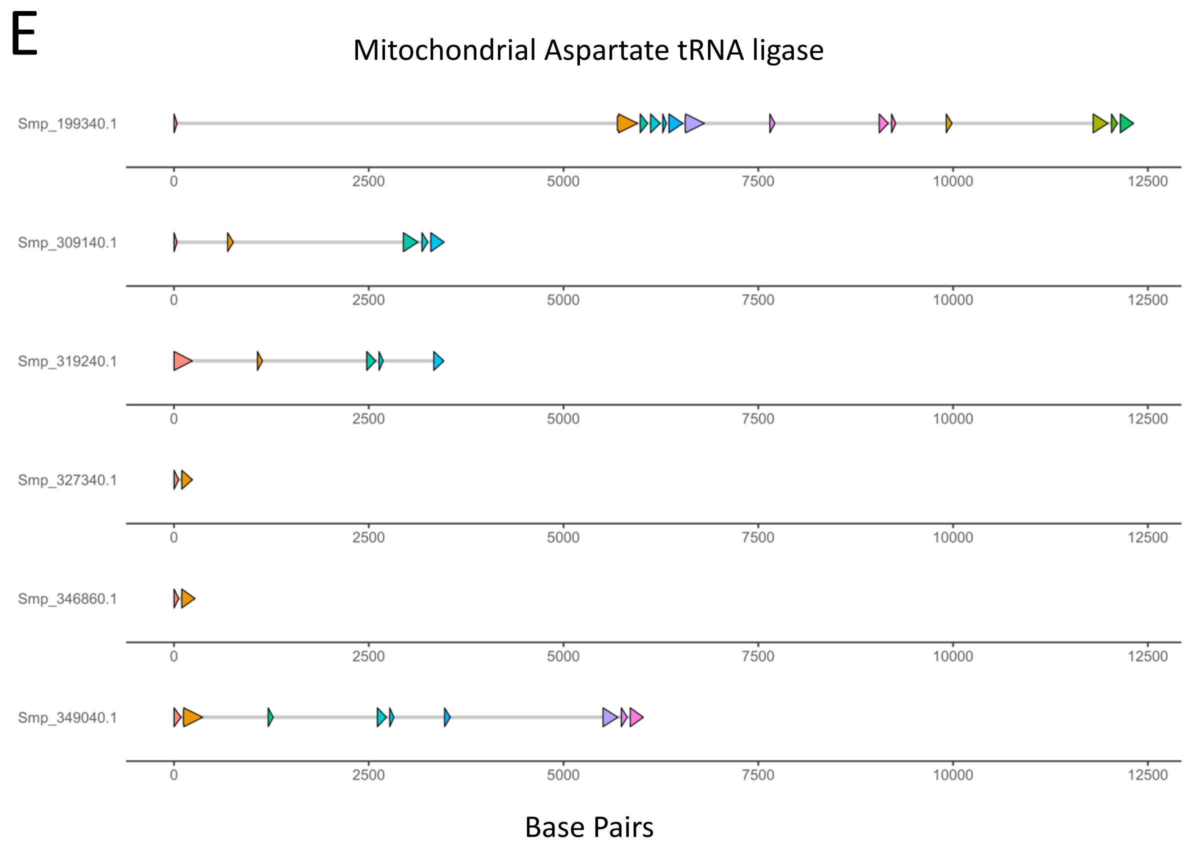

Supplementary Figure 6 - Gene models of genes that form the large subtelomeric segmentally duplicated gene clusters. (A) Hypothetical protein, **(B)** Conserved oligomeric golgi subunit 4, **(C)** Endoglycoceramidase, **(D)** Annexin, **(E)** Mitochondrial aspartate tRNA ligase clusters respectively. Arrows indicate exons (only coding sequences, excludes UTRs) for each gene, coloured by exon number except for the case of the hypothetical protein cluster. Seven genes (marked with asterisks) are lacking the canonical (as defined by the 39 "full length" gene models) first and second exons. These models were aligned and coloured relative to the third exon (for example Smp_349650.1).
