# Supplementary material for: Subtelomeric plasticity contributes to gene family expansion in the human parasitic flatworm Schistosoma mansoni
Source: BMC Genomics. 2024 Feb 27;25:217. doi: 10.1186/s12864-024-10032-8 (PMC10900676; doi:10.1186/s12864-024-10032-8)
Supplement: Supplementary file 9 — Additional file 9: Supplementary file 3. [file 12864_2024_10032_MOESM9_ESM.pdf]

V9_V10 Comparison

Toby Brann

2023-12-01

Supplementary File to Demonstrate Similiarity Between Pre-Release used in Brann et al. and the V10 Gene Annotations for S. mansoni

#Excluding mitochondrial genes, pre-release version 9.5 has 9909 genes and V10 has 9908 genes (as defined by the third column of the GFF). #These files share 9884 genes (coordinates provided in the supplementary and in comparison), the difference between these files largely coming from 19 entries that are re annotated / removed ncRNAs on the W chromosome.

LHS = V9.5, RHS = V10

Similarities & Differences

#Removing genes on the sex chromosomes, there is a median gene length change of the same gene between the two annotations of 0bp and at each border, a median base pair difference of +5bp. This indicates that most genes are being shifted in a similar pattern and direction in relation to the genome as opposed to the gene annotations themselves being changed. As we were polling segmental duplications upwards of 20kbp in length and later clustering the overlapping genes by amino acid identity, a median difference of five base pairs will functionally render a genes inclusion in the subtelomeric segmental duplications unchanged.

#Comparing just the lengths of the genes (as opposed to the shifts of the genes), we can see a median gene length change of 0bp.

```
##This is a comparison of genes from V9.5 and V10
##also provided as a supplementary (Supplementary Table 3)
path<-"/Users/tobybrann/Documents/Subtelomeres/manuscript/Reviewers/v9_v10_comp/V9.5_V10_comp.bed"
comparison <- read.delim(file=path,sep="\t",header=FALSE)

nosex_comparison <- comparison %>% subset(V5!="SM_V10_Z" & V5!="SM_V10_WSR")

##Median Gene Border Change
LHS_median<-median(nosex_comparison$V6-nosex_comparison$V2)

RHS_median<-median(nosex_comparison$V7-nosex_comparison$V3)

LHS_median
```

```
## [1] 5
```

```r
RHS_median
```

```
## [1] 5
```

```r
##Median Gene Length Change
Len_median<-median((nosex_comparison$V3-nosex_comparison$V2)-(nosex_comparison$V7-nosex_comparison$V6))

Len_median
```

```
## [1] 0
```

##Additional analysis to compute transformation of subtelomere coordinates between V9.5 and V10

#Take the genes within a given subtelomere, compare the coordinates of the gene in V9.5 and V10 #Median value for the shift of the genes per subtelomere will give us a correction factor, to shift the current subtelomere coordinates by

```r
###This is a bed file of the subtelomere coordinates provided within the manuscript
path<-"/Users/tobybrann/Documents/Subtelomeres/manuscript/V2/Figure_1/content/subtel_v3.bed"
subtels <- read.delim(file=path,sep="\t",header=FALSE)

corrs_df <- data.frame(chrom1=NA, chrom2=NA, corr=NA)[numeric(0), ]

for(row in 1:nrow(subtels)){
  subtel_q <- subtels[row,]
  temp <- data.frame(chrom=NA, LHS=NA, RHS=NA)[numeric(0), ]
  for(gene_row in 1:nrow(comparison)){
    chr<-comparison[gene_row,1]
    LHS<-comparison[gene_row,2]
    RHS<-comparison[gene_row,3]
    if(subtel_q$V1==chr & RHS >= subtel_q$V2 & LHS <= subtel_q$V3){
      temp<-rbind(temp,comparison[gene_row,])
    }
  }
  #Temp now has the intersect, we then process it
  chr_1 <- temp[1,1]
  chr_2 <- temp[1,5]
  LHS<-temp[,6]-temp[,2]
  RHS<-temp[,7]-temp[,3]
  val<-median(c(LHS,RHS))
  corrs_df <- rbind(corrs_df,c(chr_1,chr_2,val))
}

colnames(corrs_df)[1] <- "Chr_1"
colnames(corrs_df)[2] <- "Chr_2"
colnames(corrs_df)[3] <- "Correction Factor"

corrs_df
```

```
##         Chr_1    Chr_2 Correction Factor
## 1     SM_V9_1 SM_V10_1                 1
## 2     SM_V9_1 SM_V10_1                12
```

```
## 3     SM_V9_2 SM_V10_2                 1
## 4     SM_V9_2 SM_V10_2                16
## 5     SM_V9_3 SM_V10_3                 0
## 6     SM_V9_3 SM_V10_3                 7
## 7     SM_V9_4 SM_V10_4                 0
## 8     SM_V9_4 SM_V10_4                 9
## 9     SM_V9_5 SM_V10_5                 0
## 10    SM_V9_6 SM_V10_6                 0
## 11    SM_V9_6 SM_V10_6                 5
## 12    SM_V9_7 SM_V10_7                 0
## 13    SM_V9_7 SM_V10_7                 0
## 14 SM_V9_PAR1 SM_V10_Z                 1
## 15 SM_V9_PAR2 SM_V10_Z          43743328
```
